# A direct method to solve optimal knots of B-spline curves: An application for non-uniform B-spline curves fitting

**DOI:** 10.1371/journal.pone.0173857

**Published:** 2017-03-20

**Authors:** Van Than Dung, Tegoeh Tjahjowidodo

**Affiliations:** School of Mechanical and Aerospace Engineering, Nanyang Technological University, Singapore; Pennsylvania State University, UNITED STATES

## Abstract

B-spline functions are widely used in many industrial applications such as computer graphic representations, computer aided design, computer aided manufacturing, computer numerical control, etc. Recently, there exist some demands, e.g. in reverse engineering (RE) area, to employ B-spline curves for non-trivial cases that include curves with discontinuous points, cusps or turning points from the sampled data. The most challenging task in these cases is in the identification of the number of knots and their respective locations in non-uniform space in the most efficient computational cost. This paper presents a new strategy for fitting any forms of curve by B-spline functions via local algorithm. A new two-step method for fast knot calculation is proposed. In the first step, the data is split using a bisecting method with predetermined allowable error to obtain coarse knots. Secondly, the knots are optimized, for both locations and continuity levels, by employing a non-linear least squares technique. The B-spline function is, therefore, obtained by solving the ordinary least squares problem. The performance of the proposed method is validated by using various numerical experimental data, with and without simulated noise, which were generated by a B-spline function and deterministic parametric functions. This paper also discusses the benchmarking of the proposed method to the existing methods in literature. The proposed method is shown to be able to reconstruct B-spline functions from sampled data within acceptable tolerance. It is also shown that, the proposed method can be applied for fitting any types of curves ranging from smooth ones to discontinuous ones. In addition, the method does not require excessive computational cost, which allows it to be used in automatic reverse engineering applications.

## 1. Introduction

Piecewise polynomial (*pp*) functions are extensively used in many applications, such in the approximation of a complex function, data regression or data compression and in computing technology due to its simplicity and good properties. There are a few ways to represent a piecewise polynomial function from an explicit to an implicit form in Bezier or B-spline curve. The most well-known piecewise polynomial function is, perhaps, in a spline form. The spline, especially in the form of B-spline, can easily capture various functions from continuous curves to discontinuous ones.

The use of piecewise polynomial to approximate or to fit a complex function or a given data set became a popular research topic in 1970s to 1990s. The research interests commonly focused on finding the best smooth *pp* functions to represent complex functions or sampled data. Recently, there are some needs in reverse engineering applications to employ *pp* functions for representing not only smooth curves, but also curves with non-trivial cases, i.e. curves with discontinuous points, kink points, cusps or turning points from the measured data. In literature, the most common way to represent a curve with non-trivial points is by using a B-spline function.

In almost all fitting applications, to identify a B-spline function, the data is split to find the knots (breaking points or end points of each function in a piecewise function). Subsequently, a least square method is applied to calculate control points to fully determine B-spline functions. Alternatively, the curve fitting problem is formulated as a non-linear optimization problem of the knots and control points. However, the optimization problem commonly leads to a multimodal case, which results in local optima. This is the most challenging work in data fitting with B-spline. In a case when the data forms a smooth curve, many existing methods are available to solve the knots without any problems. But when the curves contain non-trivial cases (discontinuous points, kink points, cusps, turning points), the optimization process is commonly approached by using artificial algorithms, which is inspired from biological systems such as genetic algorithm, neural network or artificial immune system etc. However, these approaches usually require excessive computational time.

In this paper, we present a new method for B-spline fitting based on the combination of the knot insertion for identifying coarse knots and a local non-linear optimization to optimally identify the knot positions and continuity levels (multiple-knot). The proposed method can be applied also for non-trivial cases. The working principle of the method can be described as follows. First the data is subdivided using a bisecting method and the subset data are fitted by a single-piece B-spline with pre-defined error bound (fitting error tolerance). Based on the coarse knots as results from bisecting step, we then optimize the locations and continuity levels at the knots respectively by solving the non-linear least squares problems. The knots are subsequently used for calculating the control points in the ordinary least squares fitting to obtain the spline curves.

Some benefits are identified from the proposed method: *i*) the method can be used to automatically/semi-automatically fit a given data that will result in a B-spline function based on the estimation of the error bound of the given data, *ii*) multiple knots are naturally obtained by optimization process, which means that the proposed method can capture the curves with non-trivial cases. *iii*) the method takes the advantage of the computational speed from the bisecting technique that is used for data segmentation and Gauss-Newton method in solving a non-linear least square equation for optimal knot identification. In this method, the B-spline is obtained through a single pass method, which results in a fast computational time without sacrificing the accuracy. These advantages make the method to be potential to be used in RE applications which can give the exact solution if the data is sampled from B-spline functions.

The paper is organized as follows: section 2 reviews the existing work in literature, while section 3 presents the detail of proposed method. Section 4 details the strategy for automatic/semi-automatic fitting of a given data with B-spline functions. Section 5 presents some examples and benchmarking results to some existing methods in the literature. Some conclusions and discussions are drawn in section 6.

## 2. State of the art

In data fitting by spline, a knot vector commonly is defined in advance. Subsequently, the control points are identified based on the minimization of a least squared error between the data points and the function. Knots are usually chosen in uniform space or by Chebyshev points [1] or based on the change of radius of curvature [2]. However, uniform spaced knots might result in an overshooting problem when the curves contain non-trivial cases e.g. turning points, cusps, kink points, discontinuous points or inhomogeneous smooth curves. In order to overcome the problem, a non-uniform knot space (free knots) is introduced. Unfortunately, optimizing the number of knot and their respective locations in a non-uniform space is a challenging problem as it is computational costly.

A common way in determining the free knots is by predetermining initial knots, followed by a specific method to modify the knots, e.g. shifting knot locations and increasing or decreasing the number of knots. We can roughly categorize the approaches into two classes. The first class is started by predefining a small number of knots (usually without interior knots), and subsequently, new knots are inserted until the fitted curve satisfies a certain criterion. In the second class, denser knots are pre-determined with a lot of redundant knots. A subset of knots is then selected from the initial one by eliminating less essential knots. The remaining knots after the elimination process are the final knots for spline fitting.

In the knot insertion class, knots are usually obtained through a bisecting method. The method scans the data from left to right and utilizes local algorithm to identify the largest subinterval of data that can be represented by a polynomial function or a parametric function without violating a certain criterion. There exists some notable methods in the literature for the local fitting functions and the selection criterion. Grove et al. [3] employed a Bezier curve to regress the local data and quantify the fitting error of the mean square error of the fitted curve to determine the knots. Rice [4, 5] used polynomial function to treat the local data and to quantify the fitting error by predetermined error tolerance based on a selectable norm and the method is intended for function approximation. Ichida et al. [6] also used the bisecting method, but instead of using a fitting error tolerance, they suggested to use Trend criterion to automatically fit a given data by a cubic piecewise polynomial. Tjahjowidodo et al. [7] used parallel algorithm for bisecting the second derivative of the data to identity a linear piecewise function in determining the knot using a cubic spline fitting. Plass et al. [8] used dynamic programming to subdivide the data for identifying knots for parametric cubic spline fitting. Park et al. [9] employed dominant points instead of the direct use of knots in B-spline fitting problem and, subsequently, the knots are obtained by back transforming the dominant points. In the paper, they employed the insertion algorithm to add more dominant points until the fitted curve satisfies the predetermined error bounded tolerance. In addition, there are also some methods in literature that are based on knot insertion such as in [10, 11].

In the second class, where the algorithm starts with denser knots, different methods can be found in literature on how the initial knots are created. Lyche et al. [12, 13] used all sampled data points as candidates for the initial knots for knot removal strategy. He et al. [14] used wavelet transform to identify locations of high frequency points and put the knots at those locations to generate initial knots for knot removal process. Kang et al. [15] used a jumping distance of the third derivative (in case of cubic spline) to select the initial knots via sparse optimization through the application of Lasso optimization. Another method that is also based on the third derivative was discussed in [16]. Yuan et al. [17] used a set of multi-resolution basis functions with Lasso optimization to select the basis functions, which can compose the given data and subsequently creates the initial knots from the selected basis function set.

Apart from the two aforementioned methods in determining the knot locations, there also exists some approaches by employing optimization processes. Optimization approach is shown to be superior in the identification of the knots location, which leads to high fitting quality. However, as a price for high quality fitting results, this approach suffers from long computational time as all optimization tools for solving non-linear problems involve some iterative processes. In order to reduce the computational cost, the number of knots needs to be predetermined in advance. We can roughly classify the optimization tools into two categories i.e. the stochastic approach and deterministic approach.

In the deterministic approach, Schwetlick et al. [18] used Gauss-Newton method to solve a non-linear least square problem for knots identification. Randrianarivony et al. [19] employed Levenberg-Marquardt method to solve the same problem, which will (only) result in local optimum knot locations. In finding global optimum knots, Beliakov [20] employed the cutting angle method for fitting the global free knots spline.

On the other hand, the stochastic approach has been used in various studies. The Adaptive Free-Knot Splines (AFKS) by Miyata and Shen [21, 22] used evolution algorithm to find the optimum knots, Zhao et al. [23] employed Estimated of Distribution Algorithm (EDA), Genetic Algorithm [24] by Sarfraz et al., while Gálvez et al. [25] used Elitist clonal selection algorithm and Particle Swarm [25] for selection of knots. The Artificial immune system is also used by Ülker [26] for free knot placement.

In order to reduce the computational cost while keeping the optimal knots (local optimum knots) in the B-spline fitting process, this paper presents a method that will combine the traditional bisecting method for coarsely identifying the knots location and deterministic optimization process based on Gauss-Newton method for solving the optimal knots by the local algorithm. The details of the proposed method are given in section 3.

## 3. Local algorithm for free knots placement based on bisecting method

A *p*-degree B-spline is given as:

$$S\left(t\right)=\sum_{i=0}^{m-p-1}N_{i,p}\left(t\right)P_{i\;}$$ (1)

where *P*_*i*_ represents the *i*^*th*^ control point and *N*_*i*,*p*_(*t*) is the *i*^*th*^ *p*-degree B-spline basis function which is defined based on sequential knot vector $\text{Z}={\left\{\zeta_{j}\right\}}_{0}^{m}$ (*ζ*_0_ = *ζ*_1_ = … = *ζ*_*p*_ < *ζ*_*p*+1_ ≤ *ζ*_*p*+2_ ≤…≤ *ζ*_*m*−*p*‒1_ < *ζ*_*m*−*p*_ = *ζ*_*m*−*p*+1_ = … = *ζ*_*m*_). A *p* degree B-spline basis function *N*_*i*,*p*_(*t*) is defined in a recursive series [1]:

$$\begin{array}{ll} N_{i,0}\left(t\right) & =\{\begin{array}{ll} 1 & if\;\zeta_{i}\leq t<\zeta_{i+1}\\ 0 & otherwise \end{array}\\ N_{i,j}\left(t\right) & =\frac{t-\zeta_{i}}{\zeta_{i+j}-\zeta_{i}}N_{i,j-1}\left(t\right)+\frac{\zeta_{i+j+1}-t}{\zeta_{i+j+1}-\zeta_{i}}N_{i+1,j-1}\left(t\right) \end{array}$$ (2)

As stated above, the knot vector is a non-decreasing sequence. The first and the last (*p* + 1) knots are identical, which refer to the boundary condition of a B-spline curve. The knots from *ζ*_*p*+1_ to *ζ*_*m*−*p*‒1_ correspond to the interior knots which can be single or multiple-knots. If a knot *ζ*_*i*_ has *η* times of multiple, the spline curve at that corresponding knot location would be continuous to *C*^*k*^ with (*k* = *p* − *η*). This is a relevant property to employ B-spline to represent a non-trivial case, i.e. kink points, discontinuous points or turning points.

### 3.1 Methodology

A B-spline curve can also be used to fit a given set of data. After a proper optimization process, a spline curve in Eq (1) is fully defined if the control points *P*_*i*_ and the basis functions *N*_*i*,*p*_ are well defined. From Eqs (1) and (2) it is observed that the spline curve has a linear relationship with the control points, but *S*(*t*) is non-linear to the knot vector Z.

The common approach to identify the fitted B-spline function is by using the least square method with the following cost function:

$$\text{min}\sum_{j=1}^{n}{\left(\hat{S}\left(t_{j}\right)-Q_{j}\right)}^{2}$$ (3)

where $Q_{i}=\left(x_{j},y_{j},\ldots \right){|}_{j=1}^{n}$ is the measured *j*^*th*^ data point.

The aforementioned optimization problem is linear if the knot vector Z is predefined, where the control points can be solved in a straightforward way. The simplest way to predetermine the knot vector, Z, is by defining it uniformly. This approach can well approximate the curve if the curve is smooth. However, in a case of non-homogeneous curves or curves with non-trivial points, this approach tends to result in overshooting, thus it cannot satisfactorily fit the data. To overcome the problem, non-uniform knots with multiple knots are required. However, finding non-uniform knot is becoming a non-trivial problem. Another approach to solve the optimization problem (3) is to treat the knot vector Z as a variable. But because of non-linear searching space and multimodal property, the problem usually results in local optima.

Let us consider a case of one-piece *p*-degree B-spine that is defined in an interval [*a*, *b*] and has the following knot vector: $\text{Z}=\underset{p+1}{\underset{︸}{a,\ldots ,a}},\underset{p+1}{\underset{︸}{b,\ldots ,b}}$. To identify a one-piece fitting B-spline for a given data set, we only need to solve the least square Eq (3) to determine the control points.

**Fig 1:**
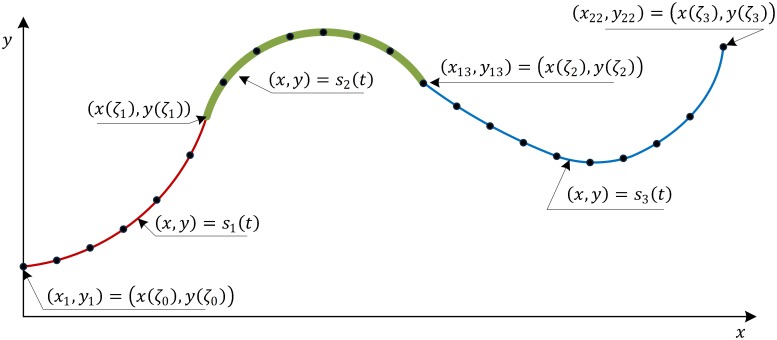
Sampled data from a piecewise polynomial function.

Methodology—Given a set of noisy sampled data from a B-spline function $Q_{i}=\left(x_{j},y_{j}\right){|}_{j=1}^{n}$, where (*x*_*j*_, *y*_*j*_) = ***S***(*t*_*j*_) + ***e***_*j*_, and ***e***_*j*_ is random error; the B-spline function ***S***(*t*) is going to be reconstructed from the measured data. Fig 1 shows a sampled case where ***S***(*t*) has three member functions *s*_1_(*t*), *s*_2_(*t*) and *s*_3_(*t*). The sampled data comprises 22 points indexed from 1 to 22. Commonly, to reconstruct the function *S*(*t*), we have to identify the three member functions i.e. *s*_1_(*t*), *s*_2_(*t*) and *s*_3_(*t*) and its breaking points Z = [*ζ*_1_, *ζ*_2_]. We can easily identify all the member functions if the data is correctly segmented, i.e. the data is split into three subintervals [(*x*_1_, *y*_1_),…,(*x*_6_, *y*_6_)], [(*x*_7_, *y*_7_),…,(*x*_13_, *y*_13_)] and [(*x*_14_, *y*_14_),…,(*x*_22_, *y*_22_)]. The interior knots can be identified by solving intersecting points of each pair of piecewise member functions if we assume that the function itself is *C*° continuity. In a case of noisy sampled data, we can only find the approximation of each member function and the knot vector, respectively.

As stated above, the most critical part in fitting data with a *pp* function is to split the data to the exact subintervals, i.e. finding the knots of the estimated *pp* function. There are three common approaches to solve the problem. The first approach is by choosing a first subinterval and identifying the first member function based on the selected subinterval. Subsequently, the subinterval is gradually expanded to the right side until the fitted member function reaches a certain criterion. The process has to be carried out iteratively until the last subinterval. This method is quite efficient but it requires high computational cost. To boost up the subdivision procedure, the data is usually split by using the bisecting method, which will be detailed in the following subsection. The second method is to guess the number and location of the knots based on a certain criterion such as uniform distribution, Chebyshev points or change in radius of curvature etc. This method tends to give good results in a case when the fitted curve is smooth. However, in a case when the curve contains non-trivial points, this method usually does not give a satisfactory result, which is due to oscillation/overshoot near the non-trivial points. The last approach is to formulate the knots to an optimal problem and using mathematical tools to find the optimal results. Unfortunately, this optimization problem is multimodal and usually results in local optima.

This paper, in the first place, presents the bisecting method for subdividing the input data. Unlike some existing methods in the literature that result in the knots without optimizing their locations [3], the knots are optimized to identify the locations and continuity levels. Multiple knots are simultaneously identified by using the optimization process.

In any fitting cases when data is subjected to noise, the member functions of the *pp* function are merely the approximation of the true ones. Therefore, the joining point of every two subsequent piecewise members will hardly coincide to the true knot. To identify the exact knots/multiple knots, we employ a two-piece B-spline function, ${\hat{S}}_{i}\left(t\right)$, to fit every pair of adjoining fitted member functions, *s*_*i*_(*t*) and *s*_*i*+1_(*t*), from bisecting step. The optimal knot/multiple-knot *ζ*_*i*_ is the solution of the non-linear least square problem (3).

**Fig 2:**
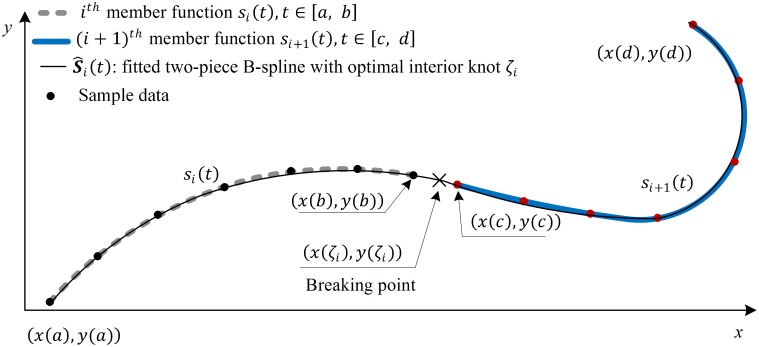
Optimal knot solving.

Fig 2 illustrates the approach in finding the optimal breaking point of a pair of member functions using B-spline. Two subsequent member function datasets separated by bisecting step *s*_*i*_, *s*_*i*+1_ are used to construct a two-piece B-spline ${\hat{S}}_{i}\left(t\right)$ by solving the optimal knot *ζ*_*i*_ of the two-piece B-spline. The continuity level *C*^*k*^ of the knot or multiple knot *η* (where *η* = *p* − *k*) is derived by comparing the fitting errors of the fitted B-splines when the interior knot $\underset{\eta }{\underset{︸}{\zeta_{i},\ldots ,\zeta_{i}}}$ is treated as multifold, i.e. *η* = 1, 2,…, *p* + 1. The details are given in subsection 3.2.4.

### 3.2 Free knots placement

There are various approaches to identify a knot vector for a B-spline fitting. In this paper, we employ bisecting method for determining the knot vector based on local algorithm followed by the knot optimization.

#### 3.2.1 Serial bisecting method for data splitting

**Fig 3:**
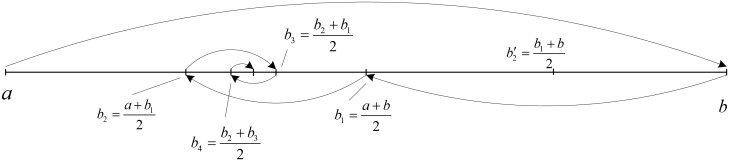
Bisecting method for subdivision data in knot identifying process.

The bisecting method is also used as a tool for identifying a knot vector in [3–6, 10]. The proposed bisecting method in this paper has similar principle to that of the method in [3], except that in this process we use a single piece B-spline to fit the data. Fig 3 illustrates the working principle of the bisecting method in splitting the data for knot placement. The objective is to find the largest interval [*a*, *b*_*n*_] ∈ [*a*, *b*] in which the fitted single-piece B-spline still comply the error bound criterion (see section 3.2.3 for details). This is an iterative process, which starts by examining the hypothesis if the fitted single piece B-spline curve can be defined from all the data of the searching interval [*a*, *b*] without violating the error bound criterion. If the hypothesis is satisfied, then the process will be terminated and if it is not, the searching interval is now shrunk to $\left[a,b_{1}=\frac{a+b}{2}\right]$.

At the second step, the new interval is examined with the similar hypothesis. If it is satisfied, the searching interval will be expanded to $\left[a,b_{2}^{\prime }=\frac{b_{1}+b}{2}\right]$, otherwise, the searching interval is reduced further into $\left[a,b_{2}=\frac{a+b_{1}}{2}\right]$. The procedures are executed repetitively until the searching interval cannot be extended anymore. The algorithm in S1 Appendix is the implementation of the serial bisecting method for rough identification of the knots.

#### 3.2.2 Parallel bisecting method

The serial bisecting method works with all data *Q*_*i*_ to find the first spline member function *s*_1_. However, if the data size is very large, the serial bisecting method might run out of memory and it also take a long computational time to evaluate the fitted function. Furthermore, the serial bisection is a sequential process, i.e. it cannot take advantages of the recent computational systems, which is capable of highly parallel processing on multicore processors. In this paper, we propose a new parallel bisecting method that is developed from our previous work [7] to overcome the limitation of the serial bisecting method. The parallel bisecting method runs faster than the serial one when the data is large and it overcomes out-of-memory problems, since it only processes the data partially. This subsection will illustrate the proposed method, while the performance will be presented later in section 5.

**Fig 4:**
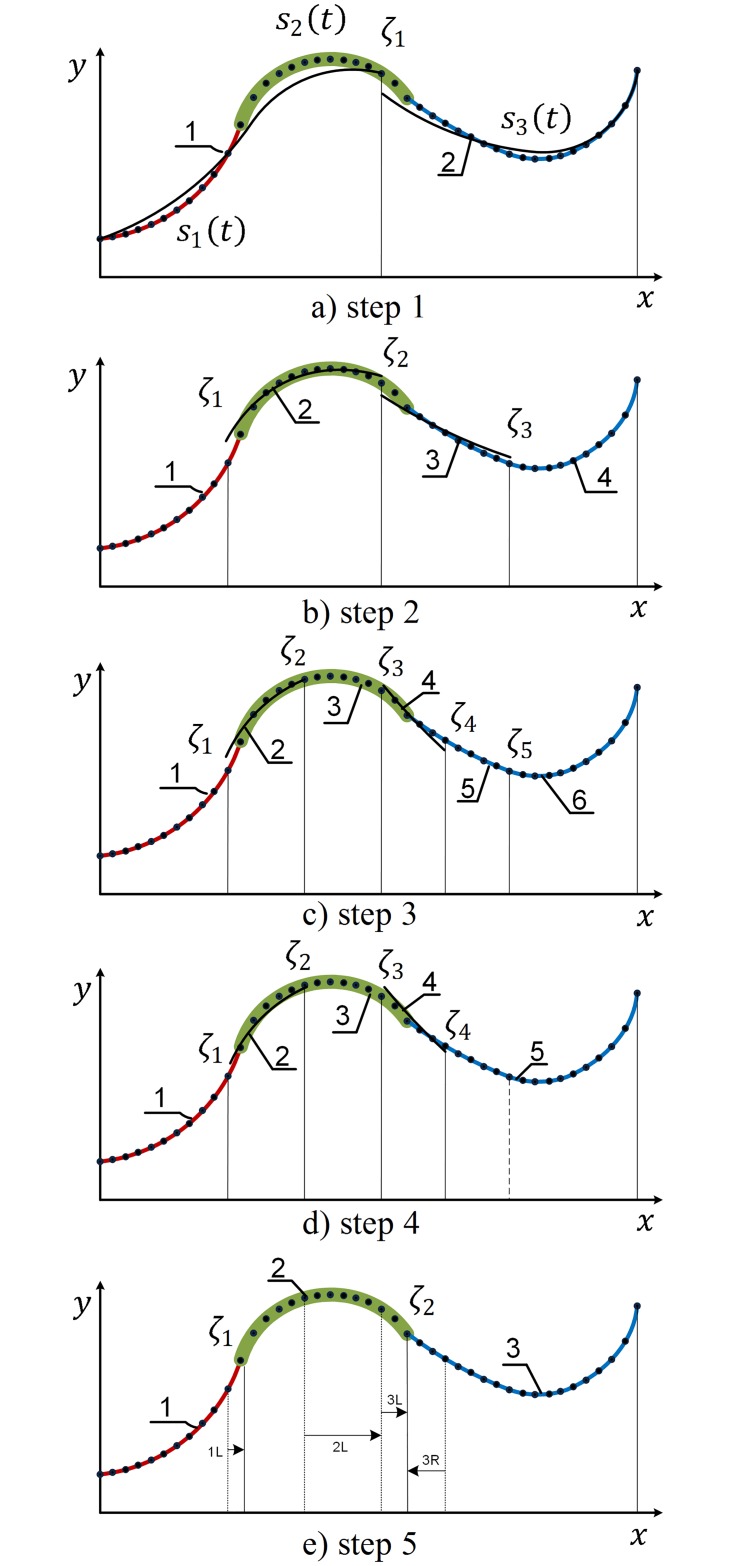
Parallel bisecting. a) Start at 2 spline pieces, b) first step, c) second step, d) joining test e) shifting.

The serial bisecting method is initiated by separating the data from the left side to the right side, while the parallel approach is started by separating data at a pre-desired number of pieces. With a proper coding, this can speed up the method by parallel programming. Fig 4 illustrates the working principle of parallel bisecting by taking the example of Fig 1. Please note that the B-spline function *S*(*t*) consists of three member functions: *s*_1_(*t*), *s*_2_(*t*) and *s*_3_(*t*). Unlike the serial method, the number of spline pieces initially is freely selected. In this example, the procedure starts by splitting *S*(*t*) into two half pieces as shown in Fig 4a. Both pieces are concurrently half-split into 4 pieces (Fig 4b). Each of the four new subsets is subsequently fitted by using one-piece B-spline and the fitting error is evaluated. If the errors are smaller than a control threshold, the pieces will remain, otherwise, they will be half-split again. Let the first and last pieces in Fig 4b pass the test and two remaining do not as illustrated in Fig 4c. Therefore, we have to split the second and third pieces further. At this step, the new pieces (2, 3, 4, and 5) have to undergo the fitting test again. At the end of step 3, for example, the two pieces (2, 4) do not pass the test, but we cannot subdivide them further because of insufficient number of samples on those pieces (we will refer pieces, such as piece 2 and 4 that have data less than 2(*p* + 1), as “small piece”). The temporary knot vector obtained at this step might contain few redundant knots such as knot *ζ*_2_, *ζ*_3_ and *ζ*_5_.

To remove the redundant knots, we need to check every two consecutive pieces, that pass the error test, whether they belong to a single piece. If the two pieces are confirmed from a single piece, they will be merged together. Because of this “joining process”, the knot *ζ*_5_ is eliminated in Fig 4d. It is noted that the joining process does not treat the two redundant knots (*ζ*_2_, *ζ*_3_) because pieces 2 and 4 have not passed the error test yet. The knots (*ζ*_2_, *ζ*_3_) and the identified knots (*ζ*_1_, *ζ*_4_) will be treated in next step.

In this last step, all the pieces will go through a “shifting procedure”. The main idea of the shifting procedure is to expand the large pieces and to reduce small pieces’ size towards the small pieces’ location. In the first small piece (piece 2 in Fig 4d), the first knot *ζ*_1_ separating the large piece 1 (Fig 4d) and the small piece 2 is shifted to the right side (knot *ζ*_1_ is shifted toward piece 2) by a serial bisecting process (1L) (1L: means the knot #1 is shifted from left to right by a serial bisecting process from left side.) to a new position as illustration in Fig 4e. The second knot *ζ*_2_ that separates small piece 2 and piece 3 in Fig 4d is then considered for shifting process. Because piece 3 is also small, the second knot *ζ*_2_ remains. Subsequently, the second small piece (piece 3) is considered. The second knot *ζ*_2_ is shifted to the right by a serial bisection procedure 2L to a new position coincide to with the third knot *ζ*_3_ as illustration in Fig 4e, and the second knot *ζ*_2_ is eliminated consequently. The third knot *ζ*_3_ is not shift to the left because the right piece (piece 4) is also small and as a result, the piece 3 is eliminated. The last shifting process will consider the last small piece (piece 4). The knot *ζ*_3_ is shifted to the right by a left-to-right serial bisection 3L to its new position and the knot *ζ*_4_ is shifted to the left 3R by a right-to-left serial bisecting process to the same position with knot *ζ*_3_ (knot *ζ*_2_ in Fig 4e). The piece 4 is, therefore, eliminated and the shifting process is accomplished. The knot vector is now obtained and the parallel bisecting process is terminated. The details in implementing the algorithm are given in S2 Appendix.

#### 3.2.3 Error bound of fitted B-spline functions

In order to decide whether a fitted function can represent the given data, any norms to calculate the fitting error can be used and, subsequently, it is benchmarked to a certain threshold as a control factor. Referring to the *l*-norm that is defined as:

$${\left\|S\left(t\right)-\hat{S}\left(t\right)\right\|}_{l}={\left(\int_{a}^{b}{\left|S\left(t\right)-\hat{S}\left(t\right)\right|}^{p}\right)}^{\frac{1}{l}}$$ (4)

We employ maximum norm 2 error as a criterion for data splitting. The error is defined as:

$$E\left(\hat{S}\left(t\right)\right)=\text{max}\left(\left|Q_{j}-\hat{S}\left(t_{j}\right)\right|\right)$$ (5)

(Eq 5) can be rewritten in the matrix form as $E=\text{max}\left(\sqrt{\text{sum}\left(\left(S-\hat{S}\right)⊙\left(S-\hat{S}\right),2\right)}\right)$, where *S* = [*Q*_1_, *Q*_2_,…,*Q*_*n*_]^*T*^ is the given data vector, $\hat{S}={\left[\hat{S}\left(t_{1}\right),\;\hat{S}\left(t_{2}\right),\ldots ,\hat{S}\left(t_{n}\right)\right]}^{T}$ is the fitted data vector, ⊙ represents element-wise multiplication operator and sum(_, 2) means summation of row elements.

The proposed method guarantees that the maximum error of a fitted curve will always be smaller than the control threshold in each fitted local B-spline. However, in practice, when the maximum error is much smaller than the noise level, the fitted curves will tend to be over-fitted. On the contrary, if the control threshold is much larger than the noise level, it usually results in under-fitted curves that fail to capture the trend of the curves.

#### 3.2.4 Knot optimization

This subsection deals with a pair of consecutive one-piece B-spline datasets from the bisecting process to find the best fitted two-piece spline function. The main task is to find optimal knot and its multiple (continuity level), respectively. In the first part, an investigation on the effect of the knot location against the fitting error function is carried out to show the characteristic of the optimal knot. In the second part, a typical deterministic optimal solver method, namely Gauss-Newton, is employed to find the optimal knot. The last part deals with the selection of the proper multiple from the few optimal knot cases.

a) Optimal interior knot of two-piece splines

Given a set of two-piece B-spline data, we have to find the optimal connection point and its continuity level which can recover the ground truth of two-piece B-spline. The first question we need to answer is “does the smallest fitting error appear when the connecting point and its continuity is set the same as its ground truth?” Answering the question will give us a hint to find the optimal knot for the two-piece B-spline.

**Fig 5:**
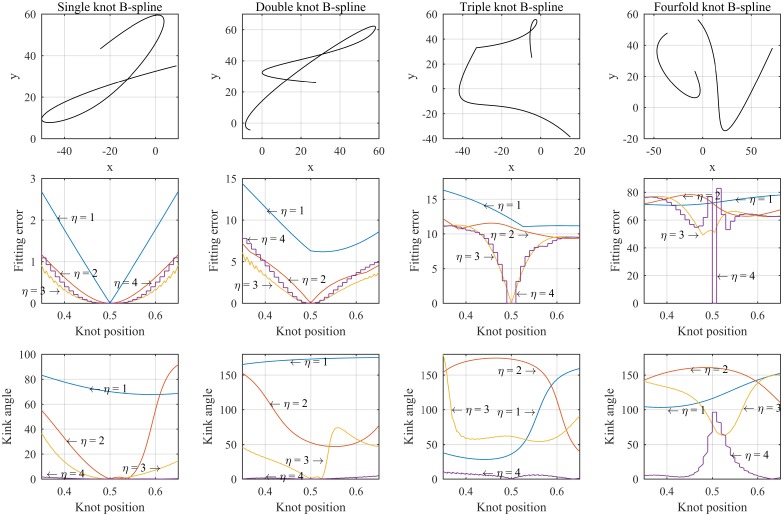
Interior knot location versus fitting error of two-piece B-spline, top panels: Two-piece spline curves, middle panels: Fitting error versus interior knot location, bottom panels: Joining kink angel versus interior knot position.

Fig 5 illustrates four cases of cubic two-piece splines that have interior knots at *t* = 0.5 (*t* = 0..1). The spline curves are shown in the first top four panels. The first B-spline has a single knot and the second has double knot, the third has triple knot and fourfold knot case for the last one. All cases are tested with different types of knot multiplication (single *η* = 1, double *η* = 2, triple *η* = 3 and fourfold *η* = 4) and the middle row panels show the fitting errors when knots vary from 0.35 to 0.65. The last four panels at the bottom illustrate the joining kink angles at the knots. We will discuss about the joining kink angle in part c) of this subsection.

When the knot is set as a fourfold, the error plots for all B-spline cases appear like staircase functions. Is that observation always true?

**Theorem 3.1** *Let ${\left\{Q_{i}\right\}}_{i=1}^{n}={\left\{Q_{i}\right\}}_{i=1}^{n1}\cup {\left\{Q_{i}\right\}}_{i=n1+1}^{n}$ be a set of data sampled from a two-piece B-spline of p degrees* $S\left(t\right)=\{\begin{array}{c} s_{1}\left(t\right)\;if\;\zeta_{s0}\leq t<\zeta \\ s_{2}\left(t\right)\;if\;\zeta \leq t<\zeta_{s1} \end{array}$ *where ${\left\{Q_{i}\right\}}_{i=1}^{n1}\in s_{1}\left(t\right)$, ${\left\{Q_{i}\right\}}_{i=n1+1}^{n}\in s_{2}\left(t\right)$ and n*_1_ > (*p* + 1), (*n*–*n*_1_) > (*p* + 1). *Given ζ is a discontinuity interior knot ((p+1)-fold knot) of the fitted B-spline function $\hat{S}\left(t\right)$. Then, the fitting error $E={max}_{i}\left(\sqrt{{\left(Q_{i}-\hat{S}\left(t_{i}\right)\right)}^{2}}\right)$ is a piecewise constant function (staircase function) of ζ*.

**Fig 6:**
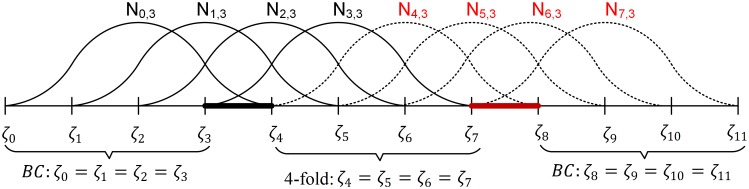
Knots and basis functions of discontinuous two-piece cubic B-spline (*p* = 3).

**Proof:** Based on the definition of B-spline, a two-pieces *p*-degree B-spline, which is discontinuous at its interior knot *ζ* ((*p*+1)-fold knot), consists of 3(*p* + 1) knots $\zeta_{i}{|}_{i=0}^{3p+2}$, 2(*p* + 1) basis functions $N_{i,p}{|}_{i=0}^{2p+1}$, and 2(*p* + 1) control points $P_{i}{|}_{i=0}^{2p+1}$ respectively. The knots are separated into three groups of identity knots *ζ*_0_ = *ζ*_1_ = … = *ζ*_*p*_ = *ζ*_*s*0_, *ζ*_*p*+1_ = *ζ*_*p*+2_ = … = *ζ*_2*p*+1_ = *ζ* and *ζ*_2*p*+2_ = *ζ*_2*p*+3_ = … = *ζ*_3*p*+2_ = *ζ*_*s*1_. The basis function *N*_*i*,*p*_ is defined in between the knots *ζ*_*i*_ and *ζ*_*i*+*p*_. Fig 6 illustrates knots and basis function of a discontinuous two-piece cubic B-spline (*p* = 3) as an example.

Let *f*_1_(*t*), *f*_2_(*t*) be *p*-degree polynomials, which are the best fit by least square method of the datasets ${\left\{Q_{i}\right\}}_{i=1}^{m}$ and ${\left\{Q_{i}\right\}}_{i=m+1}^{n}$ respectively.

We have

$$\begin{array}{ll} f_{1} & =\left[1,\;t,\;t^{2},\ldots ,t^{p}\right]{\left[\alpha_{0},\;\alpha_{1},\ldots ,\alpha_{p}\right]}^{T}\\ f_{2} & =\left[1,\;t,\;t^{2},\ldots ,t^{p}\right]{\left[\beta_{0},\;\beta_{1},\ldots ,\beta_{p}\right]}^{T} \end{array}$$ (6)

where ${\left[\alpha_{0},\;\alpha_{1},\ldots ,\alpha_{p}\right]}^{T}={\left({\left[\begin{array}{cc} \begin{array}{cc} 1 & t_{1}\\ 1 & t_{2} \end{array} & \begin{array}{cc} \ldots & t_{1}^{p}\\ \ldots & t_{2}^{p} \end{array}\\ \begin{array}{cc} \vdots & \vdots \\ 1 & t_{m} \end{array} & \begin{array}{cc} \ddots & \vdots \\ \ldots & t_{m}^{p} \end{array} \end{array}\right]}^{T}\left[\begin{array}{cc} \begin{array}{cc} 1 & t_{1}\\ 1 & t_{2} \end{array} & \begin{array}{cc} \ldots & t_{1}^{p}\\ \ldots & t_{2}^{p} \end{array}\\ \begin{array}{cc} \vdots & \vdots \\ 1 & t_{m} \end{array} & \begin{array}{cc} \ddots & \vdots \\ \ldots & t_{m}^{p} \end{array} \end{array}\right]\right)}^{-1}{\left[\begin{array}{cc} \begin{array}{cc} 1 & t_{1}\\ 1 & t_{2} \end{array} & \begin{array}{cc} \ldots & t_{1}^{p}\\ \ldots & t_{2}^{p} \end{array}\\ \begin{array}{cc} \vdots & \vdots \\ 1 & t_{m} \end{array} & \begin{array}{cc} \ddots & \vdots \\ \ldots & t_{m}^{p} \end{array} \end{array}\right]}^{T}\left[\begin{array}{c} \begin{array}{c} Q_{1}\\ Q_{2} \end{array}\\ \begin{array}{c} \vdots \\ Q_{m} \end{array} \end{array}\right]$ and

$${\left[\beta_{0},\;\beta_{1},\ldots ,\beta_{p}\right]}^{T}={\left({\left[\begin{array}{cc} \begin{array}{cc} 1 & t_{m+1}\\ 1 & t_{m+2} \end{array} & \begin{array}{cc} \ldots & t_{m+1}^{p}\\ \ldots & t_{m+2}^{p} \end{array}\\ \begin{array}{cc} \vdots & \vdots \\ 1 & t_{n} \end{array} & \begin{array}{cc} \ddots & \vdots \\ \ldots & t_{n}^{p} \end{array} \end{array}\right]}^{T}\left[\begin{array}{cc} \begin{array}{cc} 1 & t_{m+1}\\ 1 & t_{m+2} \end{array} & \begin{array}{cc} \ldots & t_{m+1}^{p}\\ \ldots & t_{m+2}^{p} \end{array}\\ \begin{array}{cc} \vdots & \vdots \\ 1 & t_{n} \end{array} & \begin{array}{cc} \ddots & \vdots \\ \ldots & t_{n}^{p} \end{array} \end{array}\right]\right)}^{-1}{\left[\begin{array}{cc} \begin{array}{cc} 1 & t_{m+1}\\ 1 & t_{m+2} \end{array} & \begin{array}{cc} \ldots & t_{m+1}^{p}\\ \ldots & t_{m+2}^{p} \end{array}\\ \begin{array}{cc} \vdots & \vdots \\ 1 & t_{n} \end{array} & \begin{array}{cc} \ddots & \vdots \\ \ldots & t_{n}^{p} \end{array} \end{array}\right]}^{T}\left[\begin{array}{c} \begin{array}{c} Q_{m+1}\\ Q_{m+2} \end{array}\\ \begin{array}{c} \vdots \\ Q_{n} \end{array} \end{array}\right].$$

The fitted B-spline is defined as

$$\hat{S}\left(t\right)=\{\begin{array}{c} {\hat{s}}_{1}\left(t\right)=\sum_{i=0}^{p}N_{i,p}P_{i}\;if\;\zeta_{s0}\leq t<\zeta \\ {\hat{s}}_{2}\left(t\right)=\sum_{i=p+1}^{2p+1}N_{i,p}P_{i}\;if\;\zeta \leq t<\zeta_{s1} \end{array}$$ (7)

∀*ζ*: *t*_*m*_ < *ζ* ≤ *t*_*m*+1_, where *t*_*m*_ and *t*_*m*+1_ are parametric of the sample point *Q*_*m*_ and *Q*_*m*+1_, and *m* ≥ (*p* + 1) and (*n* − *m*) ≥ (*p* + 1) respectively. The data is subdivided into two subsets ${\left\{Q_{i}\right\}}_{i=1}^{m}$ and ${\left\{Q_{i}\right\}}_{i=m+1}^{n}$.

The fitted B-spline can be rewritten in a matrix form as

$$\begin{array}{lll} {\hat{s}}_{1}\left(t\right) & =\left[1,\;t,\;\ldots ,t^{p}\right]\left[\begin{array}{cc} \begin{array}{cc} a_{00} & a_{10}\\ a_{01} & a_{11} \end{array} & \begin{array}{cc} \ldots & a_{p0}\\ \ldots & a_{p1} \end{array}\\ \begin{array}{cc} \vdots & \vdots \\ a_{0p} & a_{1p} \end{array} & \begin{array}{cc} \ddots & \vdots \\ \ldots & a_{pp} \end{array} \end{array}\right]\left[\begin{array}{c} \begin{array}{c} P_{0}\\ P_{1} \end{array}\\ \begin{array}{c} \vdots \\ P_{p} \end{array} \end{array}\right] & =TAP_{I}\\ {\hat{s}}_{2}\left(t\right) & =\left[1,\;t,\;\ldots ,t^{p}\right]\left[\begin{array}{cc} \begin{array}{cc} b_{00} & b_{10}\\ b_{01} & b_{11} \end{array} & \begin{array}{cc} \ldots & b_{p0}\\ \ldots & b_{p1} \end{array}\\ \begin{array}{cc} \vdots & \vdots \\ b_{0p} & b_{1p} \end{array} & \begin{array}{cc} \ddots & \vdots \\ \ldots & b_{pp} \end{array} \end{array}\right]\left[\begin{array}{c} \begin{array}{c} P_{p+1}\\ P_{p+2} \end{array}\\ \begin{array}{c} \vdots \\ P_{2p+1} \end{array} \end{array}\right] & =TBP_{II} \end{array}$$ (8)

where *P*_*I*_ = *A*^−1^[*α*_0_, *α*_1_,…,*α*_*p*_]^*T*^ and *P*_*II*_ = *B*^−1^[*β*_0_, *β*_1_,…,*β*_*p*_]^*T*^.

Because the polynomials ${\hat{s}}_{1}\left(t\right)$ and ${\hat{s}}_{2}\left(t\right)$ are the solution of the least square method for the two subsets ${\left\{Q_{i}\right\}}_{i=1}^{m}$ and ${\left\{Q_{i}\right\}}_{i=m+1}^{n}$, and the matrices *A* and *B* have rank (*p* + 1), while matrices *P*_*I*_ and *P*_*II*_ are freely defined, therefore $\{\begin{array}{l} {\hat{s}}_{1}\left(t\right)=f_{1}\left(t\right)\\ {\hat{s}}_{2}\left(t\right)=f_{2}\left(t\right) \end{array}$. Because fitting error $E=max_{i}\left(\sqrt{{\left(Q_{i}-\hat{S}\left(t_{i}\right)\right)}^{2}}\right)$ is constant for ∀*ζ*: *t*_*m*_ < *ζ* ≤ *t*_*m*+1_, therefore, the fitting error *E* is a piecewise constant function.

**Corollary 3.1**: *The ground truth knot ζ of a two-piece B-spline S*(*t*) *is located in between two subsequent samples Q*_*m*_ *and Q*_*m*+1_, *with the fitting error E of the discontinuity two-piece B-spline $\hat{S}\left(t\right)$ is equal to zero*.

**Proof**: Let *ζ* be a discontinuity ((*p* + 1)-fold) knot, we have *t*_*m*_ < *ζ* ≤ *t*_*m*+1_. The data is separated into two datasets ${\left\{Q_{i}\right\}}_{i=1}^{m}$ and ${\left\{Q_{i}\right\}}_{i=m+1}^{n}$. Because ${\left\{Q_{i}\right\}}_{i=1}^{m}\in s_{1}\left(t\right)$ and ${\left\{Q_{i}\right\}}_{i=m+1}^{n}\in s_{2}\left(t\right)$ then ${\hat{s}}_{1}\left(t\right)=f_{1}\left(t\right)=s_{1}\left(t\right)$ and ${\hat{s}}_{2}\left(t\right)=f_{2}\left(t\right)=s_{2}\left(t\right)$.

Then the fitting error $E=max_{i}\left(\sqrt{{\left(Q_{i}-\hat{S}\left(t_{i}\right)\right)}^{2}}\right)\;=0$.

**Corollary 3.2**: *If the original two-piece B-spline S*(*t*) *is discontinuous (p + 1)-fold at its interior knot, the ground truth knot ζ cannot be recovered by evaluating the fitting error E of the fitted B-spline* $\hat{S}\left(t\right)$ *using gradient method*.

**Proof**: As shown in **Corollary 3.1**, the fitting error is *E* = 0: ∀ *ζ* ∈ (*t*_*m*_, *t*_*m*+1_]. We cannot apply the gradient method to find the optimal knot.

The **Corollary 3.1** provides us a key to narrow the region in searching the optimal knot for the other multiple (continuity level) cases. As shown in Fig 5, the error functions of all cases might have many local minima except for the single knot case. We can also see that within a subsequent sample, the error functions in all cases will have only one local minimum. Therefore, any deterministic non-linear solvers can be employed to find the optimal knot. In this paper, we use Gauss-Newton method to find the optimal knots for non-discontinuous cases. The Gauss-Newton method is detailed in the next part of this subsection.

At this point, the question whether the fitting error will be the smallest when the knot is set the same as its ground truth, as stated at the beginning of this subsection is answered by **Corollary 3.1**. The smallest fitting error will not only occur when the interior knot (both position and multiplication) is set at its ground truth but also when the knot is set close to its ground truth position in discontinuous case. However, due to the computational error, it is very hard to get the optimal point with no error. This gives us the answer that the discontinuous case will usually exhibit the smallest error in practice.

b) Gauss-Newton method to solve non-linear least square problems

Recalling the least square problem (3), this subsection discusses the solution of the optimization problem for a special case when the fitted B-spline has only two pieces, i.e. one breaking point. The data is obtained by sampling two adjoining fitted local B-splines $\;{\hat{S}}_{i}\left(t\right),\;{\hat{S}}_{i+1}\left(t\right)$ which are the results from the bisecting procedure. We will optimize the location of the knot *ζ* and its continuity *C*^*k*^, by examining the multiple knot case from single to (*p* + 1) folds (the detail was given in Fig 2).

A two-piece B-spline which is defined in the interval [*T*_*a*_, *T*_*d*_], will have the following knot vector $Ζ\left(T_{a},\zeta ,T_{d}\right)=\underset{p+1}{\underset{︸}{\langle T_{a},T_{a},\ldots ,T_{a}}},\underset{\eta \;=\;p-k}{\underset{︸}{\zeta ,\ldots ,\zeta }},\underset{p+1}{\underset{︸}{T_{d},T_{d},\ldots ,T_{d}\rangle }}$ where: *p* is the degree of B-spline, *k* is the level of continouity *C*^*k*^, *k* = −1, 0,…, (*p* − 1).

Eq (3), thus, can be rewritten in a matrix form as

$${\text{min}}_{i}\sum {\left(S\left(t_{i}\right)-Q_{i}\right)}^{2}=\text{min}{\left(X-NP_{x}\right)}^{\text{T}}\left(X-NP_{x}\right)\;+\;\text{min}{\left(Y-NP_{y}\right)}^{\text{T}}\left(Y-NP_{y}\right)$$ (9)

where: $X={\left[\begin{array}{c} x_{1}\\ x_{2}\\ \vdots \\ x_{n} \end{array}\right]}_{1\times n}$, $Y={\left[\begin{array}{c} y_{1}\\ y_{2}\\ \vdots \\ y_{n} \end{array}\right]}_{1\times n}$, $N={\left[\begin{array}{cc} N_{l\times \left(p+1\right)}^{1} & 0\\ 0 & N_{v\times \left(p+1\right)}^{2} \end{array}\right]}_{n\times \left(2p+1-k\right)}$ and

$$N_{l\times \left(p+1\right)}^{1}=\left[\begin{array}{ccc} N_{0,p}\left(t_{1}\right) & \begin{array}{cc} N_{1,p}\left(t_{1}\right) & \cdots \end{array} & N_{p,p}\left(t_{1}\right)\\ \begin{array}{c} N_{0,p}\left(t_{2}\right)\\ \vdots \end{array} & \begin{array}{c} \begin{array}{cc} N_{1,p}\left(t_{2}\right) & \cdots \end{array}\\ \ddots \end{array} & \begin{array}{c} N_{p,p}\left(t_{2}\right)\\ \vdots \end{array}\\ N_{0,p}\left(t_{l}\right) & \begin{array}{cc} N_{1,p}\left(t_{l}\right) & \cdots \end{array} & N_{p,p}\left(t_{l}\right) \end{array}\right],$$

$$N_{v\times \left(p+1\right)}^{2}=\left[\begin{array}{ccc} N_{p-k,p}\left(t_{l+1}\right) & \begin{array}{cc} N_{p-k+1,p}\left(t_{l+1}\right) & \cdots \end{array} & N_{\left(2p-k\right),p}\left(t_{l+1}\right)\\ \begin{array}{c} N_{p-k,p}\left(t_{l+2}\right)\\ \vdots \end{array} & \begin{array}{c} \begin{array}{cc} N_{p-k+1,p}\left(t_{l+2}\right) & \cdots \end{array}\\ \ddots \end{array} & \begin{array}{c} N_{\left(2p-k\right),p}\left(t_{l+2}\right)\\ \vdots \end{array}\\ N_{p-k,p}\left(t_{n}\right) & \begin{array}{cc} N_{p-k+1,p}\left(t_{n}\right) & \cdots \end{array} & N_{\left(2p-k\right),p}\left(t_{n}\right) \end{array}\right].$$

Please note that the matrix of the basis function, *N*, is in block diagonal form because a spline piece is defined in (*p+1*) basis functions.

In [18], the authors suggested that the control point vector *P*_*x*_, *P*_*y*_ should be reformulated in Moore-Penrose pseudo-inverse as:

$$\begin{array}{cc} P_{x}={\left[\begin{array}{c} P_{x1}\\ P_{x2}\\ \vdots \\ P_{x\left(2p+1-k\right)} \end{array}\right]}_{\left(2p+1-k\right)\times 1}=N^{+}X,\; & P_{y}={\left[\begin{array}{c} P_{y1}\\ P_{y2}\\ \vdots \\ P_{y\left(2p+1-k\right)} \end{array}\right]}_{\left(2p+1-k\right)\times 1}=N^{+}Y \end{array}$$

where *N*^+^ = pinv(*N*).

Moore-Penrose pseudo-inverse can generally be evaluated by using the Singular Value Decomposition (SVD). In case of redundant data, pseudo-inverse can be evaluated using a normal least squares approach, which requires less computational cost compared to that of the SVD method. This motivates us to employ least square method to solve the control points.

Optimization problem (9) is rewritten as

$${\text{min}}_{i}\sum {\left(S\left(t_{i}\right)-Q_{i}\right)}^{2}=\text{min}(G_{x}^{T}G_{x}+G_{y}^{T}G_{y})=\text{min}G^{T}G$$ (10)

where: *G*_*x*_ = *X* − *NP*_*x*_ = (*g*_*x*1_, *g*_*x*2_,…,*g*_*xn*_)^*T*^, and *G*_*y*_ = *Y*–*NP*_*y*_ = (*g*_*y*1_, *g*_*y*2_,…,*g*_*yn*_)^*T*^, with *P*_*x*_ = argmin(*X*–*NP*_*x*_) and *P*_*y*_ = argmin(*Y*–*NP*_*y*_) are the solutions of the least squares problems. *G* = (*g*_1_, *g*_2_,…, *g*_*n*_)^*T*^, with $g_{i}=\sqrt{g_{xi}^{2}+g_{yi}^{2}}$.

To solve the non-linear least squares Eq (10) using the Gauss-Newton method, the first derivative of the function *G* needs to be obtained. Based on the computational complexity of the B-spline, we obtain the first derivative of *G* by numerical method as

$$\frac{dG\left(\zeta \right)}{d\zeta }=G^{\prime }\left(\zeta \right)\approx \frac{G\left(\zeta +h\right)-G\left(\zeta \right)}{h}$$ (11)

where: $h=\sqrt{macheps}$ is the step of approximating the first derivative.

The knot *ζ*^*s*+1^ at step *s*+1 of the iterative procedure will be calculated by

$$\zeta^{s+1}=\zeta^{s}-{\left(G^{\prime }{\left(\zeta \right)}^{T}G^{\prime }\left(\zeta \right)\right)}^{-1}G^{\prime }{\left(\zeta \right)}^{T}G\left(\zeta \right)$$ (12)

c) Multiple knot selection

The analysis of the optimal knot position discussed in Section 3.2.4a shows that each multiple knot case has its own optimal position. The optimal knot in the discontinuity case usually provides the smallest fitting error. The multiple-knot cannot be, therefore, selected based on the fitting error itself.

Considering the definition of multiple knots, given two pieces of a B-spline of degree *p* which are connected at a *η*-multiple knot, the B-spline function is continuous to (*p* − *η*)^*th*^ derivative at the knot location. It means that the (*p*–*η* + 1)^*th*^ derivative at the knot is discontinuous. We can explore the property to select the multiple-knot for two-piece B-spines.

**Fig 7:**
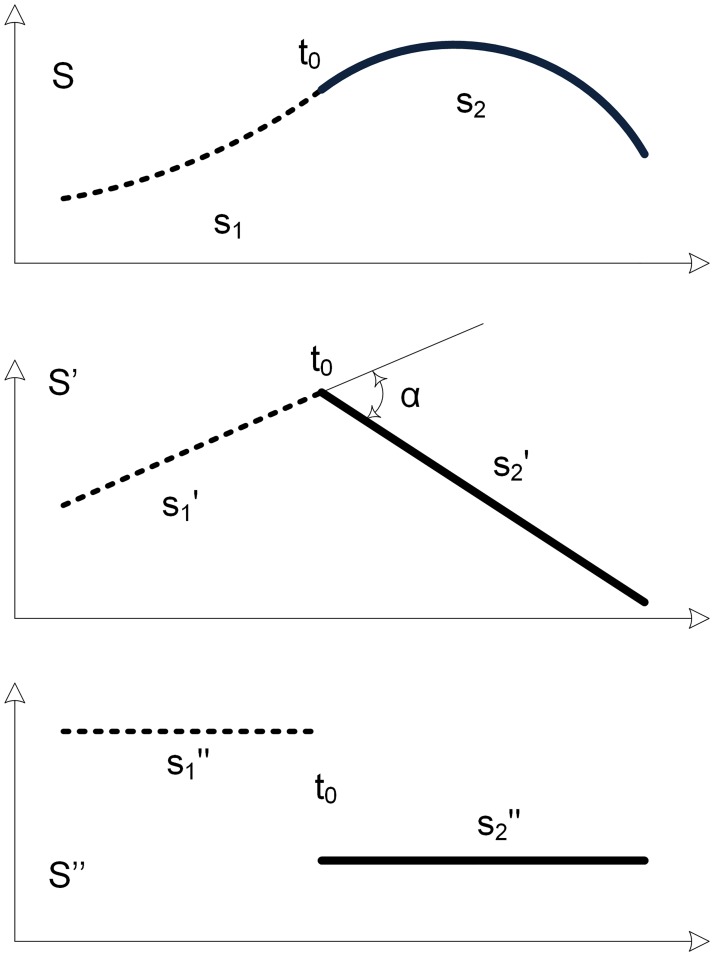
Two-piece B-spline at its derivatives.

Fig 7 illustrates a two-piece B-spline and its derivatives. Assuming that the B-spline is continuous at the knot location to its first derivative and it is discontinuous in its second derivative, we can easily see that the first derivative has a kink angle α at the knot location.

The kink angle α is calculated from

$$\text{cos}\alpha =\text{cos}\left(∠\left(\vec{s_{1}^{\prime }}\left(t_{0}\right),\vec{s_{2}^{\prime }}\left(t_{0}\right)\right)\right)=\frac{\vec{s_{1}^{\prime \prime }}\left(t_{0}\right).\vec{s_{2}^{\prime \prime }}\left(t_{0}\right)}{\left||\vec{s_{1}^{\prime \prime }}\left(t_{0}\right)||||\vec{s_{2}^{\prime \prime }}\left(t_{0}\right)|\right|}$$ (13)

where $\vec{s_{1}^{\prime }}\left(t_{0}\right),\vec{s_{2}^{\prime }}\left(t_{0}\right),\vec{s_{1}^{\prime \prime }}\left(t_{0}\right),\vec{s_{2}^{\prime \prime }}\left(t_{0}\right)$ are the vector values of the first derivative and second derivative at the knot location approaching from the left side and right side, respectively.

In general, a joining knot of a two-piece *p* degree B-spline can have (*p+1*) cases i.e. single knot to (*p+1*)-fold knot. The single knot case has a kink angle at the (*p-1*)^th^ derivative. Similarly, the double knot case has a kink angle at the (*p-2*)^th^ derivative and so on. The kink angle *α*_*η*_ of multiple interior knot *η* case is computed by Eq (14).

$$\alpha_{\eta }=\text{acos}\left(\frac{\vec{s_{1}^{\left(p-\eta +1\right)}\left(\zeta \right)}.\vec{s_{2}^{\left(p-\eta +1\right)}\left(\zeta \right)}}{\left||\vec{s_{1}^{\left(p-\eta +1\right)}\left(\zeta \right)}||||\vec{s_{2}^{\left(p-\eta +1\right)}\left(\zeta \right)}|\right|}\right)$$ (14)

**Theorem 3.2**: Let *S*(*t*) *be a p degree two-piece B-spline which has interior knot at ζ and knot multiplication η* < (*p* + 1). *Then, the kink angle of the discontinuity interior knot ((p + 1)-fold knot) of the fitted two-piece B-spline $\hat{S}\left(t\right)$ by* Eq (14) *at the interior knot position ζ is equal to zero*, *α*_p+1_(*ζ*) = 0.

**Proof:** As a result of theorem 3.1, two member functions of the fitted B-spline ${\hat{s}}_{1}\left(t\right)$ and ${\hat{s}}_{2}\left(t\right)$ is joined at the knot position *ζ*, i.e. ${\hat{s}}_{1}\left(\eta \right)={\hat{s}}_{2}\left(\eta \right)$.

The kink angle of the discontinuity case in Eq (14) now can be rewritten as:

$$\alpha_{p+1}=\text{acos}\left(\frac{\vec{s_{1}^{\left(0\right)}\left(\zeta \right)}.\vec{s_{2}^{\left(0\right)}\left(\zeta \right)}}{\left||\vec{s_{1}^{\left(0\right)}\left(\zeta \right)}||||\vec{s_{2}^{\left(0\right)}\left(\zeta \right)}|\right|}\right)=\text{acos}\left(\frac{\vec{{\hat{s}}_{1}^{\left(0\right)}\left(\zeta \right)}.\vec{{\hat{s}}_{1}^{\left(0\right)}\left(\zeta \right)}}{\left||\vec{{\hat{s}}_{1}^{\left(0\right)}\left(\zeta \right)}||||\vec{{\hat{s}}_{1}^{\left(0\right)}\left(\zeta \right)}|\right|}\right)=\text{acos}\left(1\right)=0.$$

Fig 5 shows the optimal knot and kink angle of each spline case. It is also observed that at the optimal knot (*t* = 0.5), the kink angles of the discontinuity cases follow the theorem 3.2.

Combining information of fitting error and kink angle will lead us to the true multiple knot. A multiple knot case is selected if the kink angle α of the knot is larger than a certain threshold, *α*_*min*_, and has smaller fitting error. The program in S3 Appendix will give the details of the implementation in the optimal knot solving and selecting.

## 4. A strategy for fitting of non-uniform B-spline curves

This section summarizes the proposed method for B-spline fitting. In principle, there are three steps in identifying a B-spline function. The first step is parameterization of the input data to convert the data into parametric form, which strongly affects the fitted B-spline curve quality [27]. Selection of a proper parameterization method is essential in this step. There exists some methods such as ‘Chord length’, ‘Uniformly spaced’ or ‘Centripetal’ for the parameterization method. Based on authors’ knowledge, the ‘Chord length’ method is widely used in most of cases. The second step is the estimation of the noise level of the data as a basis for determining the error bound. The last step is the application of the proposed method for knot identification and then the least square to fit the input data for identification of control points of the B-spline.

Selecting the proper maximum error (error bound) will affect the number of knots. If the selected maximum fitting error is much smaller than the noise level, the fitting curve will be over-fitted and, the fitted curve will be under-fitted otherwise. Therefore, the maximum error should be selected based on the noise level of the input data.

In short, the strategy for fitting of the given data by B-spline curve with free and multiple knots can be summarized as follows:

Step 1: Parameterizing the input data to obtain a data set (*t*_*i*_, *x*_*i*_, *y*_*i*_).
Step 2: Estimating noise level to calculate *∈*.
Step 3: Applying bisecting method to solve coarse knots.
Step 4: Optimizing the coarse knots to identify the optimal ones by solving non-linear least squares and select the optimal multiple-knot.
Step 5: Using least square method to calculate the control points of the fitted B-spline curve based on the optimal knots.
Step 6: Computing the fitted B-spline curve and plot results.

## 5. Results

The proposed method is numerically validated and the results are discussed in this section. Three distinct experimental validations with different sets of data are used. In the first experiment, datasets are sampled from B-spline functions, and in the second experiment, datasets are generated from deterministic functions. While the first two experiments consider clean data, the third one will examine the performance of the proposed method in the presence of noise. In all cases, we employ Mean Square Error $\;\left(MSE\right)=\frac{1}{N}\sum_{i=1}^{N}{\left(\left|\hat{S}\left(t_{i}\right)-Q_{i}\right|\right)}^{2}=\frac{1}{N}\sum_{i=1}^{N}\left({\left({\hat{x}}_{i}-x_{i}\right)}^{2}+{\left({\hat{y}}_{i}-y_{i}\right)}^{2}\right)$, Root Mean Square Error $(RMSE)=\sqrt{MSE}$ and Maximum Error $\left(ME\right)=\text{max}\left|\hat{S}\left(t_{i}\right)-Q_{i}\right|=\text{max}\sqrt{{\left({\hat{x}}_{i}-x_{i}\right)}^{2}+{\left({\hat{y}}_{i}-y_{i}\right)}^{2}}$ to quantify the performance of the method. In this section, we also discuss the processing speed performance of the method implemented in MATLAB 2014a environment running on a Core 2 Duo T7300 2.0GHz processor with 3GB of RAM computer.

### 5.1 Fitting data sampled from a spline function

In this section, we present the results of testing the proposed method with various B-spline data with randomly knot vector. The results are depicted in the following figures and the tables.

A spline function that was proposed by Kang et al. [15] with the same setting parameters are used. The function is generated using the interior knots listed in Table 1. The function is uniformly sampled with 1001 points (note that there exists a double knot at 0.5408).

**Table 1:** Identified interior knots.

Ground truth knots	Residual errors from proposed method
0.0439	-1.318e-13
0.0653	-5.273e-12
0.2293	-3.972e-12
0.2367	-1.771e-09
0.4821	-1.160e-10
0.4907	-2.216e-10
0.5408	-1.840e-12
0.5408	-1.840e-12
0.6209	-3.926e-12
0.7051	-1.126e-13
0.9407	-5.221e-11

**Fig 8:**
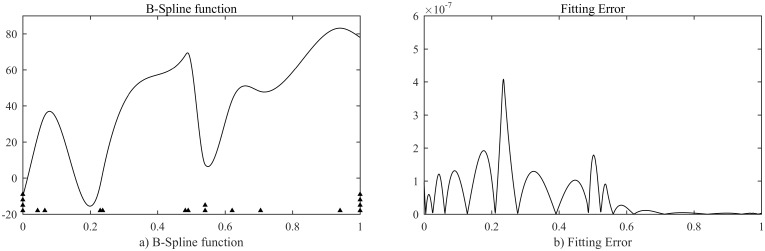
Approximation of a cubic B-spline.

Fig 8 depicts the B-spline function in the left panel with the fitting error in the right panel. The proposed method results in nearly exact multiple and position of interior knots with the residual errors are given in Table 1. The mean square error (MSE) of this fitting is 8.046e-15 that is achieved in about 0.3 second. We can see that the interior knots approximate the true ones with the maximum residual error is -1.771e-09. The errors of the calculated knots most likely are caused by the error in numerical solution in the optimization process. The details of setting parameters are listed in Table 2.

**Table 2:** Differences between Serial and Parallel bisecting.

Parameters	Serial bisection	Parallel bisection
Spline Degree	*p* = 3	*p* = 3
Control error	*∈* = 1*e* − 6	*∈* = 1*e* − 6
Minimum kink angle (degree)	*α*_*min*_ = 0.002	*α*_*min*_ = 0.002
Maximum smoothness C(k)	*k* = −1	*k* = −1
Data bisecting time (ms)	25.7	27.6
Coarse knots by bisecting step	1, **46**, 67, 231, 238, 484, 492, 542, 623, 707, 943, 1001	1, **45**, 67, 231, 238, 484, 492, 542, 623, 707, 943, 1001
Optimal interior knot residual errors	**-2.096e-14**, -4.306e-12, 6.652e-13, -2.986e-12, -8.347e-11, -8.886e-11, -1.427e-11, -1.427e-11, -5.143e-12, -7.424e-09, -3.039e-11	**-5.297e-14**, -4.306e-12, 6.652e-13, -2.986e-12, -8.347e-11, -8.886e-11, -1.427e-11, -1.427e-11,-5.143e-12, -7.424e-09, -3.039e-11

Table 2 shows the differences between serial and parallel bisection approaches. All setting parameters for the two methods are kept the same, but there is a slightly difference in the results. The coarse knots by both methods are almost the same except the second knot, where the serial method results in 46 while the counterpart results in 45 (the correct number must be 45 with *t* = 0.044). The serial method tends to shift the knot to the right because its algorithm is based on left-to-right bisection. As a result, the final optimal knot of the second knot (first interior knot) exhibits a small difference due to different start points in Gauss-Newton solving step. Furthermore, the processing time of the parallel method is a bit slower by 2ms.

In comparison to the sparse optimization method [15], the proposed method gives the MSE = 8.046e-15 while the counterpart method exhibits MSE = 3.7596e-6. It can be also highlighted that the computational time of the proposed method is about 0.3 second, while the counterpart took about 40 seconds.

For further discussion on fitting data sampled from B-spline functions, please refer to S4 Appendix.

### 5.2 Approximating deterministic functions

In this subsection, the performance of the proposed method is evaluated on parametric deterministic functions. Two different functions are used, i.e. a butterfly curve and a spur gear curve that was sampled from a 3D model generated using Solidworks. In the first case, the butterfly curve was generated using two functions [28]:

$$\{\begin{array}{c} x\left(t\right)=\text{sin}\left(t\right)\left(e^{\text{cos}\left(t\right)}-2\text{cos}\left(4t\right)-{\text{sin}}^{5}\left(\frac{t}{12}\right)\right)\\ y\left(t\right)=\text{cos}\left(t\right)\left(e^{\text{cos}\left(t\right)}-2\text{cos}\left(4t\right)-{\text{sin}}^{5}\left(\frac{t}{12}\right)\right) \end{array},\;t=\left[0,\;2\pi \right].$$

**Fig 9:**
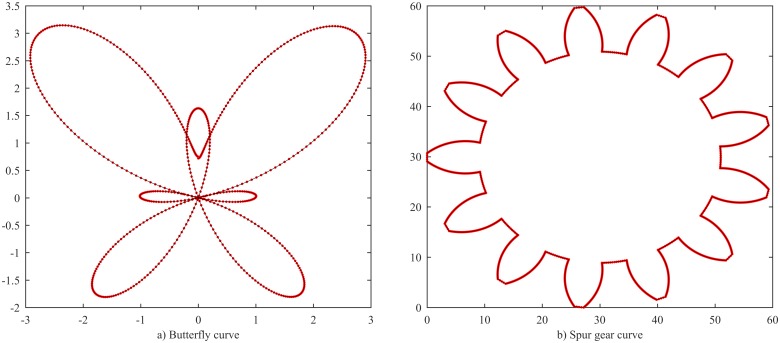
Fitted cubic splines of butterfly (left panel) and spur gear (right panel) curves. Dotted points: sample data, solid curves: fitted spline.

The butterfly function is sampled with 629 points at a uniform space *t* = 0.01. The resulting curve is illustrated in the left panel of Fig 9. Table 3 lists some different cases in setting parameters. The number of interior knots strongly depends on the control error threshold when the other parameters are kept unchanged. In the case of cubic B-spline, *p* = 3, the number of interior knots increases from 31 to 72 when the adjusted error *∈* decreases from 1e-3 to 1e-5. We also obtain the same results when the degree of spline is *p* = 2 in case 3 and *p* = 4 in case 4. Even the optimization is set to find optimal multiple-knots from 1 to (*p*+1) fold, the algorithm only results in single-knots.

**Table 3:** Some selected cases of the fitting butterfly curve.

Case No.	Fitting parameters				Results			
	Degree*p*	Max error *ϵ*	Min kink angle *α*_*min*_	Continuity C^k^	Max fitting error (ME)	Mean squared error (MSE)	No. interior knot (K)	No. pieces (Ψ)
1	3	1e-3	15	-1	0.0019	1.5948e-6	31 single-knots	32
2	3	1e-5	15	-1	8.8161e-5	1.3989e-9	72 single-knots	73
3	2	1e-5	15	-1	1.9663e-4	8.5433e-9	126 single-knots, 1 triple-knot	128
4	4	2e-6	15	-1	7.5484e-5	1.0302e-9	60 single-knots	61

Similarly, on the right panel of the Fig 9 and Table 4, the results of the fitted spline for the spur gear curve are presented. The data is sampled from a spur gear geometric data (with module of 4 and 13 teeth). The spur gear curve is generated by sampling a 3D model created using Solidworks (it was digitized by converting the drawing to stereolithography format (STL) to create triangular mesh). The data is sampled in non-uniformly space with 1612 points as shown in the right panel of Fig 9. Table 4 provides detail comparison between two bisecting methods. It can be seen that the processing time for the parallel bisecting took about 100 milliseconds (100, 105 and 107) and it is less sensitive to the change of the number of spline pieces. On the contrary, the serial bisection needs more time and highly depends on the number of spline pieces (130,197 and 269 milliseconds). The total processing time heavily depends on optimal knot solving. It does not only depend on the number of interior knots but also depends on initial start points for Gauss-Newton solving.

**Table 4:** Some cases of fitting the spur gear curve.

Case no.	1		2		3	
Fitting parameters	Serial bisecting	Parallel bisecting	Serial bisecting	Parallel bisecting	Serial bisecting	Parallel bisecting
Spline Degree *p*	3	3	3	3	2	2
Control error *∈*	1e-2	1e-2	1e-3	1e-3	1e-3	1e-3
Minimum kink angle (degree) *α*_*min*_	1	1	1	1	5	5
Maximum smoothness C(k)	-1	-1	-1	-1	-1	-1
Data bisection time (ms)	130	100	197	105	269	107
Coarse knots by bisection step	52 interior knots	52 interior knots	78 interior knots	78 interior knots	130 interior knots	181 interior knots
Multiple knot	52 Triple-knots	52 Triple-knots	52 triple-knots, 26 single-knots	52 triple-knots, 26 single-knots	78 single-knots, 52 double-knots	129 single-knots, 52 double-knots
Fitting error: MSE (ME)	2.341e-06 (0.0060)	2.421e-06 (0.0055)	1.562e-07 (0.0013)	6.099e-08 (0.0012)	7.4063e-7 (0.0018)	1.1562e-7 (8.862e-4)
Total processing time (ms)	2490	2501	3640	3715	3970	5635

As we can see, the spur gear has 52 kink points (13 teeth × 4 kink points). As listed in Table 4, the numbers of kink points are correctly defined for all cases. In the first case, the number of interior knots is equal to 52, this leads to all interior triple knots. There is a minor difference in the optimal knots obtained from the two different bisection approaches as highlighted in S1 Table. Because the data is approximated by a B-spline, the optimal knots will deviate when the input data is slightly changed. For the two remaining cases, the number of spline piece increases when the control error is decreased, but the number of multiple-knots (kink points) is the same as that in the first case while the extended knots are all single-knot.

### 5.3 Noisy data

In this subsection, we present the results of the proposed method in the presence of noise on the data. To quantify the effectiveness of the method and for benchmarking purpose, we employ three functions *φ*_1_, *φ*_2_, *φ*_3_ which were used in references [25, 29] to make a comparison with the Elitist clonal selection [29].

**Fig 10:**
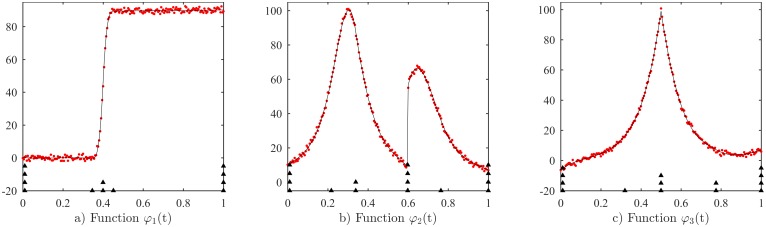
Noisy functions for benchmarking with Elitist clonal selection methods [29]. a) *φ*_1_(*t*), b) *φ*_2_(*t*), c) *φ*_3_(*t*) (dots: sample data, solid lines: fitted curve, triangle marker: knot position).

Three benchmarking functions, which are adopted form [25, 29], are used with the same set of parameters (the data is uniformly sampled with 201 points and the randomized noise is natural distribution with *μ* = 0 *and σ* = 1). Fig 10 represents the three functions and Table 5 provides the numerical results of the comparison. We can see that the results from the proposed method offers higher accuracy compared to those from the Elitist clonal selection method, while the processing time is notably faster (higher computing performance of Intel Core i7 2.6GHz, 8GB RAM is used in [29]).

**Table 5:** Average Root Mean Squared Error RMSEs and Standard Deviations (SD) for 50 simulation replications, comparison with Elitist clonal selection method (data are reproduced from Tables 2, 3 and 4 of [29]).

Functions	Proposed method		Elitist clonal selection	
	RMSE(SD)	Run time (second)	RMSE	Run time (second)
*φ*_1_(*t*)	0.4153 (0.1135)	0.275	1.06581	10.92
*φ*_2_(*t*)	0.5272 (0.1360)	0.229	0.87377	21.71
*φ*_3_(*t*)	0.3909 (0.0859)	0.147	0.89368	24.87

## 6. Conclusion

In this article, we have proposed a new method for optimal knot calculation in a B-spline fitting based on a local B-spline fitting technique that is capable for non-uniform knot cases. The working principle of the method is based on employing the bisecting method with a specific error bound as a criterion to find the best fitted single piece B-spline for the given data and to identify the coarse knots. The coarse knots are, subsequently, optimized to identify the optimal knots. The method is proven to be able to reconstruct B-spline functions for various sampled data. In comparison to the existing methods in the literature, the method offers faster computational time that is attributed to a single pass process (referred to as a one-pass method in [6]) without sacrificing its accuracy, whereas in many cases it offers better accuracy than the existing methods. One more apparent advantage of the proposed method is that the processing time does not depend too much to the sample size. Yet, the control factor (error bound) has significant effect on the processing time. In typical applications, where the data size is smaller than 1000, the processing time is only about few seconds.

In short, as the fast processing time is the main feature of the method, it offers as a potential tool for such applications in reverse engineering, computer aided design and computer aided manufacturing.

## Supporting information

S1 Appendix Pseudo code for serial bisecting. (DOCX)

S2 Appendix Pseudo code for parallel bisecting. (DOCX)

S3 Appendix Pseudo code for optimal knot solver. (DOCX)

S4 Appendix Case examples of fitting data sampled from a spline function. (DOCX)

S1 Table Details of fitting the spur gear curve. (DOCX)

S1 File MATLAB codes for a case of fitting a B-spline generated data. (ZIP)
