# Supplementary material for: A direct method to solve optimal knots of B-spline curves: An application for non-uniform B-spline curves fitting
Source: PLoS One. 2017 Mar 20;12(3):e0173857. doi: 10.1371/journal.pone.0173857 (PMC5358887; doi:10.1371/journal.pone.0173857)
Supplement: S1 Appendix — (DOCX) [file pone.0173857.s001.docx]

# Appendix 1. Pseudo code for serial bisecting.

**Input**: data points$Q_{i}=\left( x_{i}, y_{i},\ldots\right)|_{i=1}^{n}$ , parameter$t_{i}|_{i=1}^{n}$, maximum fitting error$\epsilon$, degree of fitted B-spline$p$.

**Output**: Knot vector $Z$ with the knots coincide to the sample points, fitted local B-spline function set.

**Pseudo code for serial bisection method**:

**Initialization:**

$S=\left[ Q_{1},Q_{2},\ldots,Q_{n} \right]^{T}; T=\left[ t_{1},t_{2},\ldots, t_{n} \right]^{T};$

StartIdx = 1;

EndIdx = n;

$Z_{local}=\left( 0, \ldots,0 \right)_{1\times(2p+2)}$

$E_{max}=0$

**Loop:**

while EndIdx > StartIdx

LeftIdx = StartIdx;

RightIdx = EndIdx

while (RightIdx – LeftIdx) <= 1

if (StartIdx+p)>= *n*

break;

endif

LocalBsplineFitting();

Success = 0;

if $E_{Max}\leq\epsilon$

Success = 1;

Save temporary local spline

Save temporary $E_{max}$

endif

if Success == 1

LeftIdx = EndIdx;

else

RightIdx = EndIdx;

endif

EndIdx = floor((LeftIdx+RightIdx)/2);

endwhile

StartIdx = LeftIdx + 1;

EndIdx = *n*;

Save local spline

Save knot

endwhile

**Subfunction:**

LocalBsplineFitting(){

% Computing b-spline basis function

$Z_{local}=\left( \underset{p+1}{\underbrace{T\left( \mathrm{StartIdx} \right),\ldots, T\left( \mathrm{StartIdx} \right)}},\underset{p+1}{\underbrace{T\left( \mathrm{EndIdx} \right),\ldots, T\left( \mathrm{EndIdx} \right)}} \right)$;

$N_{mat}=\left( \begin{matrix} \begin{matrix} N_{0,p}\left( T\left( \mathrm{StartIdx} \right) \right) & N_{1,p}\left( T\left( \mathrm{StartIdx} \right) \right) \\ N_{0,p}\left( T\left( \mathrm{StartIdx}+1 \right) \right) & N_{1,p}\left( T\left( \mathrm{StartIdx}+1 \right) \right) \end{matrix} & \cdots& \begin{matrix} N_{p,p}\left( T\left( \mathrm{StartIdx} \right) \right) \\ N_{p,p}\left( T\left( \mathrm{StartIdx}+1 \right) \right) \end{matrix} \\ \vdots& \ddots& \vdots\\ \begin{matrix} N_{0,p}\left( T\left( \mathrm{EndIdx} \right) \right) & N_{1,p}\left( T\left( \mathrm{EndIdx} \right) \right) \end{matrix} & \cdots& N_{p,p}\left( T\left( \mathrm{EndIdx} \right) \right) \end{matrix} \right)$;

% identify control points for the last segment by solving equation 3.3

$P=\left( N_{mat}^{T}N_{mat} \right)^{-1}N_{mat}^{T}S$

% Computing fitting error

$E_{0}=S-N_{mat}P;$

$E^{2}= E_{0}\odot E_{0}$ ; % element-wise multiplication

$E_{Max}=\mathrm{sqrt}(\max\left( {\mathrm{sum}(E}^{2},2) \right))$;

}
