# Supplementary material for: A direct method to solve optimal knots of B-spline curves: An application for non-uniform B-spline curves fitting
Source: PLoS One. 2017 Mar 20;12(3):e0173857. doi: 10.1371/journal.pone.0173857 (PMC5358887; doi:10.1371/journal.pone.0173857)
Supplement: S2 Appendix — (DOCX) [file pone.0173857.s002.docx]

# Appendix 2: Pseudo code for parallel bisecting

**Input:** Curve dataset $S_{n\times m}$ ($S=\left\{ X_{n\times1},Y_{n\times1}, \ldots\right\}$), parameteric vector$T_{n\times1}$, number of pieces at the start Ω, fitting error$\epsilon$, degree of the fitted b-spline$p$.

**Output:** The coarse knot vector in indexing number $Z_{K}$

**Pseudo code for parallel bisection:**

**% Initializing**

$Z_{k}=1:\mathrm{round}(\frac{N-1}{\Omega-1}):n$; % initial knot vector

$dZ_{k}=\mathrm{diff}(Z_{k})$; % knot vector difference

$\Delta Z_{max}=\max dZ_{k}$; % maximum size of a piece

TestResult = ones($1,dZ_{k}$); % one array; assuming all pieces do not pass the error test.

**% Main loop**

while ($\Delta Z_{max}\geq2\left( p+1 \right)$)

**% Half split**

j = 2; $Z_{ktemp}\left( 1 \right)=Z_{k}\left( 1 \right);$

for i = 1:numel($dZ_{k})$ % for each piece

if (TestResult(i)==1)&&( $dZ_{k}\left( i \right)\geq2\left( p+1 \right)$) % half split the piece

Midpoint = round($0.5\times\left( Z_{k}\left( i+1 \right)+Z_{k}\left( i \right) \right);$

$Z_{ktemp}\left( j \right)=$ Midpoint;

$\mathrm{TestResult}_{temp}\left( j-1 \right)=1;$

j = j + 1;

$Z_{ktemp}\left( j \right)=Z_{k}\left( i+1 \right)$ ;

$\mathrm{TestResult}_{temp}\left( j-1 \right)=1;$

j = j + 1;

else % keep the piece

$Z_{ktemp}\left( j \right)=Z_{k}\left( i+1 \right)$ ;

$\mathrm{TestResult}_{temp}\left( j-1 \right)=\mathrm{TestResult}_{temp}\left( i \right);$

j = j + 1;

endif

endfor

**% Evaluate fitting error**

**-** Update new $Z_{k}, dZ_{k}, \Delta Z_{max},TestResults$ $Z_{k}=Z_{ktemp}$; $\Delta Z_{max}=ceil\left( 0.5\times\Delta Z_{max} \right)$; $\mathrm{TestResult}=\mathrm{TestResult}_{temp};$

foreach new pieces

- Fit each pieces by one-piece b-spline function.

- Evaluate fitting errors.

- Compare the fitting errors with control threshold

if $FittedError>\epsilon$

TestResult(i) = 1;

else

TestResult(i) = 0;

endif

endfor

**% Join sequential pieces**

- find pieces have just passed the error test

foreach i = found pieces

**% join with left piece**

if i ~= 1 % not the first piece

if left piece passed the error test

- Try to join two pieces, if the new piece passes the error test, the knot $Z_{k}\left( i \right)$ would be eliminated.

endif

endif

- Update date $Z_{k}$ and piece dataset

**% join with right piece**

if i ~= numel($dZ_{k})$ % not the last piece

if right piece passed the error test

- Try to join two pieces, if the new piece passes the error test, the knot $Z_{k}\left( i+1 \right)$ would be eliminated.

endif

endif

- Update date $Z_{k}$ and piece dataset

Endfor

**% Shift small pieces**

if $\Delta Z_{max}<2\left( p+1 \right)$

$dZ_{k}=\mathrm{diff}(Z_{k})$;

Smallpieces = find($dZ_{k}<2\left( p+1 \right)$);

if Smallpieces ~= null % exist small pieces

for i = 1:numel(Smallpieces)

**% expand left piece using serial bisecting method**

if i ~= 1 % not the first piece

LeftIdx = $Z_{k}(i)$;

RightIdx = $Z_{k}\left( i+1 \right)-1$;

StartIdx = $Z_{k}(i-1)$;

SerialBisectingLeft2Right();

- Update date $Z_{k}$ and piece data,

end

**% expand right piece using serial method**

if i ~= numel($dZ_{k}$) % not the last piece

if the right piece is large (not small)

LeftIdx = $Z_{k}(i)$;

RightIdx = $Z_{k}\left( i+1 \right)-1$;

EndIdx = $Z_{k}\left( i+2 \right)-1$;

SerialBisectingRight2Left();

- Update date $Z_{k}$ and piece dataset

endif

endif

endfor

break;

endif

endif

endwhile
