# Supplementary material for: A direct method to solve optimal knots of B-spline curves: An application for non-uniform B-spline curves fitting
Source: PLoS One. 2017 Mar 20;12(3):e0173857. doi: 10.1371/journal.pone.0173857 (PMC5358887; doi:10.1371/journal.pone.0173857)
Supplement: S3 Appendix — (DOCX) [file pone.0173857.s003.docx]

# Appendix 3: Pseudo code for optimal knot solver

This part summaries the algorithm to solve optimal knot of two-piece B-spline and provides the details of the algorithm.

**Input:** A set of data points of a two one-piece B-splines $\left\{ T_{d\times1},S_{d\times m} \right\}$, the starting and ending indexing of each local B-spline functions $\left[ a,b,c,d \right]$, degree of b-spline *p*, number of samples for scanning *L*, number of loop for Gauss-Newton solver *M*, minimum kink angle $\alpha_{min}$, minimum smoothness *k* and scanning all flag *ScanAllFlag*.

**Output:** Optimal knot $\zeta_{op}$ and knot multiplication $\eta$ of the optimal knot.

**Step 1: Estimating knot searching range**

- Estimating searching range for each multiple level.

for i = 1: (p + 1)

- LeftRange[i] = min(b-a-p,3) + p - i;

- RightRange[i] = min(d-c-p,3) + p - i;

endfor

- Computing step $h=\sqrt{macheps}$

**Step 2: Uniformly scan to find coarse knot location**

$\eta_{max}=p+1$ ; maximum multiple fold (discontinuity)

for j = (p + 1):-1:1

- Computing uiniform knot within the searching range.

Knotstep = (T[c+ RightRange [j]]- T[b-LeftRange[j]]) / (L-1);

StartPoint = T[max(b-LeftRange[j],a)];

StopPoint = T[min(c+ RightRange [j],d)];

if StopPoint > StartPoint

KnotSeach = StartPoint :Knotstep : StopPoint;

for i = 1:L

$\zeta$ = KnotSeach[i];

- Forming two-piece knot vector

$$Z\left( T[a],\zeta,T[d] \right)= \left\langle\underset{p+1}{\underbrace{T[a],\ldots,T[a}]},\underset{j}{\underbrace{\zeta,\ldots,\zeta}},T[\underset{p+1}{\underbrace{d],\ldots,T[d]}} \right\rangle$$

- Forming N matrix

$$N=\left[ \begin{matrix} N_{l\times\left( p+1 \right)}^{1} & 0 \\ 0 & N_{v\times\left( p+1 \right)}^{2} \end{matrix} \right]_{n\times\left( 2p+2 \right)}$$

- Computing control point P

$$P=\left( N^{T}N \right)^{-1}N^{T}S$$

- Computing maximum error

$$e=\max\left( \sqrt{sum\left( \left( S-NP \right)\odot\left( S-NP \right) ,2 \right)} \right)$$

- Computing kink angle

$$\alpha=\mathrm{acos} \left( \frac{\vec{s_{1}^{(p-j+1)}\left( \zeta\right)}.\vec{s_{2}^{(p-j+1)}\left( \zeta\right)}}{\left\| \vec{s_{1}^{(p-j+1)}\left( \zeta\right)} \right\|\left\| \vec{s_{2}^{(p-j+1)}\left( \zeta\right)} \right\|} \right)$$

- Saving the Error and angle

DisError[i] = $e$;

Alpha[i] = $\alpha$;

Endfor

if (j == p+1)

- Idx = find(DisError > (min(DisError)+1e-10)

- DisIdx = round(Idx[1]+Idx[end]);

- Error[j] = DisError[DisIdx];

- Angle[j]= Alpha[DisIdx];

- OptimalKnot[j]= KnotSeach[DisIdx];

if ~*ScanAllFlag*

- OptimalKnot[1:p] = KnotSeach[DisIdx];

- SearchRange[1:p,:] = {T[max(DisIdx -1,1)], T[min(DisIdx +1,n)]};

break;

endif

else

- Idx = find(min(DisError));

- Error[j] = DisError[Idx];

- Angle[j] = Alpha[Idx];

- OptimalKnot[j] = KnotSeach[Idx];

- SearchRange[j,:] = {T[max(Idx-1,1)], T[min(Idx+1,n)]};

endif

else

$\eta_{max}=\eta_{max}-1$;

endif

endfor

**Step 3: Optimal knot solving using Gauss-Newton method**

for j = 1: min(p, $\eta_{max}$) % for each multiple knot case

for i = 1:M % M times iteratively run

- Forming the knot vector of fitted B-spline: $Z\left( T[a],\zeta,T[d] \right)$ and $Z\left( T[a],\zeta+h,T[d] \right)$

- Computing $N\left( \zeta\right)$ and $N\left( \zeta+h \right)$matrices.

- Computing $G\left( \zeta\right)$ and $G\left( \zeta+h \right)$

- Computing $G^{'}\left( \zeta\right)$

- Saving previous step $\Delta\zeta_{0}=\Delta\zeta$

- Computing new step $\Delta\zeta= {\left( G^{'}\left( \zeta\right)^{T}G^{'}\left( \zeta\right) \right)^{-1}G}^{'}\left( \zeta\right)^{T}G\left( \zeta\right)$

- Checking non-convergent case

if ($\Delta\zeta_{0}\times\Delta\zeta<0$)

$\Delta\zeta=0.5\times\Delta\zeta$;

endif

- Saving knot location $\zeta_{0}=\zeta$

- Updating new knot location $\zeta=\zeta-\Delta\zeta$

- Saturating knot location $\zeta=\left\{ \begin{aligned} \mathrm{SearchRange}\left[ j,1 \right] if \zeta<\mathrm{SearchRange}\left[ j,1 \right] \\ \mathrm{SearchRange}\left[ j,2 \right] if \zeta>\mathrm{SearchRange}\left[ j,2 \right] \\ \zeta else \end{aligned} \right.$

- Break condition

if $\mathrm{abs}\left( \zeta-\zeta_{0} \right)<h$

cntflag = cntflag + 1;

if cntflag >= 2

break;

endif

else

cntflag = 0;

endif

endfor

- Computing maximum fitting error $ErrorMax=\max G$

- Computing kink angle

$\alpha=\mathrm{acos} \left( \frac{\vec{s_{1}^{(p-j+1)}\left( \zeta\right)}.\vec{s_{2}^{(p-j+1)}\left( \zeta\right)}}{\left\| \vec{s_{1}^{(p-j+1)}\left( \zeta\right)} \right\|\left\| \vec{s_{2}^{(p-j+1)}\left( \zeta\right)} \right\|} \right)$

- Saving fitting error, kink angle and optimal knot

$\mathrm{Error}[j]$ = $ErrorMax$;

OptimalKnot$[j]$ =$\zeta$;

Angle$\left[ j \right]=\alpha$ ;

endfor

**Step 4: Selecting continuity and optimal knot**

Returning the continuous level $k$ and optimal knot $\zeta$ satisfy the condition

$\eta=p-k$*;*

$\eta$ *=* min*(*$\eta$*,* $\eta_{max}$);

SelectedAngle = Angle[1: $\eta$] *;*

Idx = find(SelectedAngle >$\alpha_{min}$ )

if Idx ~= null

Error1 = Error[Idx];

[~,Idx1] = min(Error1);

OptimalIdx = Idx[Idx1];

- Optimal knot output: $\zeta_{op}=$ OptimalKnot[OptimalIdx];

- Multiple knot output: $\eta=$OptimalIdx;

else

[~,OptimalIdx] = max(SelectedAngle);

- Optimal knot output: $\zeta_{op}=$ OptimalKnot[OptimalIdx];

- Multiple knot output: $\eta=$OptimalIdx;

Endif

**Program explanation:**

The program processes a pair of two sequential one-piece B-spline datasets resulting from bisecting process to find the optimal knot and its continuity. The dataset contains parametric vector $T_{d\times1}$ and curve vector$S_{d\times m}$, where *n* is the number of data in the dataset and *m* is the curve dimension. The datasets have already been coarsely separated by their indexing number [*a*,*b*] and [*c*,*d*] of the first and the second spline pieces, respectively. The algorithm requires some tuning parameters, e.g. the number of uniform knots, *L*, needed in a searching process, the number of loop for Gauss-Newton solver, *M*, and a flag, namely *ScanAllFlag*, to decide the scanning process for non-discontinuous cases. The program also requires two parameters in the optimal knot selection, namely the smallest acceptable kink angle, $\alpha_{min}$, and the minimum continuity level C(*k*).

The main disadvantage of the bisecting process is the incapability of giving a good knot location in a case when the spline piece has a number of samples that is smaller than the order of the fitted curve. In a case when a spline piece has sufficient data to fully define its function, the optimal knot is usually located within two samples next to the coarse knot. Another thing needs to be considered in the estimation of the knot searching range is the condition to solve the least square problem. The least square problem would only be solved if the input data is fully or over constrained. In a case of a two-piece spline, we can easily see that “a two-piece spline is fully defined if and only if the matrix *N* is in full rank”. The condition would be satisfied if the following two conditions are satisfied. The first condition is about the total samples inside the dataset. The number of data in the dataset must be larger or equal to (*p +* $\eta)$. The second condition is related to the minimum number of samples inside a spline piece, i.e. the number of samples in single pieces must be larger or equal to the multiples $\eta$ of the interior knot.

As seen on the first part of subsection 3.2.4, if the data is sampled from a B-spline curve, we would only need to uniformly scan knots for the discontinuous case to narrow down the searching range of other multiple cases. However, in a case when the data is not originated from a B-spline curve, such as approximating a function or measured data, a uniform scan is required not only for the discontinuous case, but also for all knot multiplications. A variable *ScanAllFlag* is used to decide whether a uniform scanning is required for non-discontinuous cases. Please note that turning on the flag will take some costs in the processing time. The scanning range for non-discontinuous cases is larger than that for the discontinuous case when the number of data in a single-piece is smaller than the order of the fitted curves.

Evaluation of the uniform knots is rather straightforward. A knot is first used to form the knot vector *Z* and subsequently the basic function matrix *N* is computed. The control point vector *P* is then computed using a typical linear least square formula. The fitting error and joining kink angle is computed and saved in every step. In the discontinuous case, the smallest piecewise constant is concluded by finding the smallest error area. Due to the computational error, the lowest piecewise constant is selected with a small variation from the minimum error value (1e-10 is selected in our algorithm). As analyzed in subsection 3.2.4, the accurate knot for discontinuous cases cannot be obtained by the two-piece B-spline approach. The optimal discontinuous knot is than selected in the middle of the lowest error piecewise constant. In other non-discontinuous multiple knot cases, the searching areas are selected within one sample of the minimum points.

The next step is to find the optimal knots for non-discontinuous cases. As also analyzed in subsection 3.2.4, the error functions have only one optimal point within a sample step. Therefore, we can apply traditional gradient methods to find the optimal point. In this paper, we employ the Gauss-Newton method to solve the optimization problems. The implementation details are illustrated in step 3 of the algorithm. In Gauss-Newton method, the optimization sometime exhibits a non-convergence problem, because of its severe non-linear nature. In the algorithm, we propose a simple method by reducing the solution step by half if the sign of the solution step changed.

The last step is to decide the best multiple-knot. The best solution must satisfy two conditions: (*i*) the joining kink angle has to be larger than the input criterion, $\alpha_{min}$, and (*ii*) the fitting error has to be minimum. In the case that there are no multiple cases satisfying the first condition, we will select the multiple-knot case that has a larger joining kink angle.
