# Supplementary material for: A direct method to solve optimal knots of B-spline curves: An application for non-uniform B-spline curves fitting
Source: PLoS One. 2017 Mar 20;12(3):e0173857. doi: 10.1371/journal.pone.0173857 (PMC5358887; doi:10.1371/journal.pone.0173857)
Supplement: S4 Appendix — (DOCX) [file pone.0173857.s004.docx]

# Appendix 4: Case examples of fitting data sampled from a spline function

Table 1 provides some selected cases to explore the proposed method in fitting data from B-spline functions. A dataset has 1001 samples, which is uniformly distributed from 0 to 1. The interior knots and control points are randomly generated and the values are given in the second and third columns. Tuning parameters and fitting results are also given in the remaining columns.

**Table 1 some selected B-spline fitting cases**

| **Case No.** | **B-spline** | | | | | |
| --- | --- | --- | --- | --- | --- | --- |
|  | **Curve parameters** | | **Tuning parameters** | **Results** | | |
|  | **Interior knot** | **Control points** |  | **Knot residual errors** | **Fitting error**  **MSE (ME)** | **Processing time (s)** |
| 1 | 0.0975, 0.1270, 0.1576, **0.2785, 0.2785,** 0.6324, 0.8147, 0.9058, 0.9134, **0.9575, 0.9575,** 0.9706 | 43.5629 -21.9836; -28.1009 -37.5540; 41.7354 13.8033; 15.7865 -16.3747; 16.8415 44.5216; -9.5789 -0.9723; -8.3908 23.8681; 20.0089 -17.0458; -66.7244 17.3024; -61.3980 35.7705; -91.3120 -7.6142; -45.0084 -30.9239; 68.4833 12.9979; 54.5535 -26.2096; 5.9591 -12.3227; 10.9505 -63.8203 | $p=3,$  $\epsilon=1e-6,$  $\alpha_{min}=1, \Omega=10,$  $k=-1,$  $L=10,$  $M=10$, ScanAllFlag = 1 | -9.702e-12, -2.387e-14, -4.383e-14, **-1.443e-15, -1.443e-15,** -4.319e-14, -3.874e-13, -1.100e-09, -2.451e-09, **-2.579e-11, -2.579e-11,** -4.534e-11 | 2.1806e-14  (8.4577e-07) | 0.68 |
| 2 | 0.0159, **0.0257, 0.0257, 0.0257,** 0.1789, 0.1890, 0.2027, 0.2027, 0.5251, 0.6607, 0.8623, 0.8964, 0.9412, 0.9550, 0.9550, 0.9711, 0.9711 | 34.0669 28.8683; 49.0064 11.2947; -60.3171 75.1773; 13.9671 -27.8541; -5.5041 -94.5445; 32.9569 42.4848; 18.3631 27.8358; 6.9897 42.4366; 8.5920 62.6100; 41.5537 9.1750; 15.2572 29.0424; 22.4530 77.5639; 6.3710 -15.8599; 2.7949 -16.7661; -17.0091 -74.2350; 66.9951 -20.1932; -2.8528 -18.3535; -52.5438 -6.1148; 10.2738 28.8462; -29.6878 59.9450; 19.2656 16.2393 | $p=3,$  $\epsilon=1e-6,$  $\alpha_{min}=1, \Omega=10,$  $k=-1, L=11,$  $M=10$ ScanAllFlag = 1 | -1.804e-16, **-1.037e-13, -1.037e-13, -1.037e-13**, -1.544e-12, -3.366e-12, 2.578e-14, 2.578e-14, 2.908e-09, 6.051e-14, -2.848e-12, -3.380e-11, -1.603e-11, -5.611e-11, -5.611e-11, -1.071e-11, -1.071e-11 | 9.5370e-16 (9.0450e-08) | 0.81 |
| 3 | 0.0182, 0.0300, 0.0669, 0.0871, 0.5357, 0.5357, **0.7837, 0.7837, 0.7837, 0.7837**, 0.9861, 0.9891 | -37.1649 34.7088;-37.2243 37.4669; 29.0562 -24.6073; 93.5777 -25.5102; -16.6569 -85.1886; 6.5970 61.1681; -0.4123 81.3929; 29.4039 25.5439; 28.4196 48.1495; -46.0174 15.3203; -43.4006 -0.9348; 45.5901 35.5605; 25.8792 36.9262; -26.1438 -33.9563; 12.5146 -64.6840; 12.6804 -0.3025 | $p=3,$  $\epsilon=1e-6,$  $\alpha_{min}=5,$  $\Omega=21,$  $k=-1,$  $L=11,$  $M=10$,  ScanAllFlag = 1 | -1.388e-15, -4.703e-09, -6.273e-15, -5.770e-13, -2.232e-14, -2.232e-14, **-9.207e-06, -9.207e-06, -9.207e-06, -9.207e-06,** -3.956e-09, 3.690e-08 | 4.424e-12  (4.0622e-05) | 0.49 |
| 4 | 0.4675, 0.4675, 0.4675**,** 0.6624, 0.6992, **0.8754, 0.8778** | -29.4917 -2.7472; 32.5520 -29.5839; -27.0494 -11.3169; 30.8630 51.3881; 38.8477 -58.4587; 3.0817 -41.2673; -48.7069 -6.8617; -88.2647 21.9906; -62.3831 40.5862; 64.8031 -41.8770; 29.4571 7.5956 | $p=3, \epsilon=1e-$  $6,$  $\alpha_{min}=5,$  $\Omega=15,$  $k=-1,$  $L=10,$  $M=10$, ScanAllFlag = 1 | -2.720e-15, -2.720e-15, -2.720e-15’ -1.740e-11, -8.069e-12, **-1.610e-05, -7.973e-05** | 9.8063e-06  (0.0170) | 0.33 |
| 5 | 0.0405, 0.0654, 0.0707, 0.0894, 0.0958, 0.0958, 0.1822, 0.1822, 0.4522, 0.5978, 0.6104, 0.7016, **0.7313, 0.7319,** **0.8122, 0.8146,** 0.9039 | -15.9938 74.7236; 35.7199 18.0746; 13.8208 -3.2704; 19.0836 61.0421; 46.4001 15.3345; -16.3042 -29.7101; -0.8816 43.4445; -6.4741 6.5342; -90.4129 -36.0837; 17.4897 12.2327; 18.1252 -18.2312; 31.3901 -2.3955; -16.5180 -67.9798; -6.9273 -30.3777; 41.2263 -59.5293; -5.4938 67.0179; 32.3061 24.2833; 52.4240 4.4178; 19.2111 -16.6427; -3.0610 43.9823; -18.9611 -20.6339 | $p=3,$  $\epsilon=1e-5,$  $\alpha_{min}=5,$  $\Omega=15,$  $k=-1,$  $L=10,$  $M=10$, ScanAllFlag = 1 | -5.412e-16, -6.385e-13, -1.817e-11, -1.416e-14, -2.486e-13, -2.486e-13, -2.714e-12, -2.714e-12, -7.191e-09, -1.233e-10, -5.200e-11, -1.496e-12, **2.946e-4, -2.918e-4,** **-4.678e-06, 1.270e-05,** -8.559e-13 | 1.8054e-07  (0.0062) | 0.92 |

The first three cases deal with double, triple and fourfold-knot. As implies by the low knot residual errors, we can say that for non-discontinuous cases the method is able to estimate the knots and their multiplications satisfactory. However, for case 3, the fourfold knot indicates a relatively high residual error (9.207e-6). This is due to the case discussed in the Corollary 3.2.

In case 4, two residual errors for knots 0.8754 and 0.8778 are evidently larger compared to the rests because there are only two samples within the corresponding piece. In such cases, the serial bisection fails to perform in data separation because it needs at least (*p+*1) samples for a piece. Any error in data separation could lead to error in the computed optimal knots.

Case 5 shows another failure of the method as indicated by the high residual errors in knots 0.7313, 0.7319, 0.8122 and 0.8146. The first two knots are located within two sequential samples, which means there is no actual data within them. In such case, the method results in a double-knot at 0.73157. The knots 0.8122 and 0.8146 also fail to be optimized for the same reason as in case 4.

For all cases, the processing time heavily depends on the number of the interior knots that need to be solved. It is usually less than a second to fit the data with about 1000 samples.
