# Supplementary material for: A direct method to solve optimal knots of B-spline curves: An application for non-uniform B-spline curves fitting
Source: PLoS One. 2017 Mar 20;12(3):e0173857. doi: 10.1371/journal.pone.0173857 (PMC5358887; doi:10.1371/journal.pone.0173857)
Supplement: S1 Table — (DOCX) [file pone.0173857.s005.docx]

# S1 Table: Details of fitting the spur gear curve.

| **Case no.** | **Fitting parameters** | **Serial bisection** | **Parallel bisection** |
| --- | --- | --- | --- |
| 1 | Spline Degree | $p=3$ | $p=3$ |
|  | Control error | $\epsilon=1e-2$ | $\epsilon=1e-2$ |
|  | Minimum kink angle (degree) | $\alpha_{min}=1$ | $\alpha_{min}=1$ |
|  | Number of pieces at start | $\Omega= 1$ | $\Omega= 20$ |
|  | Maximum smoothness C(k) | $k=-1$ | $k=-1$ |
|  | Number of uniform data in scanning step | $L=10$ | $L=11$ |
|  | Number of loop in Gauss-Newton solving | $M=10$ | $M=10$ |
|  | Scanning knot | ScanAllFlag = 1 | ScanAllFlag = 1 |
|  | Data bisection time (ms) | 130 | 100 |
|  | Coarse knots by bisection step | 8,**62**,66,120,132,**186**,190,**244**,256,**310**,314,368,**380**,**434**,438,492,504,**558**,562,**616**,628,**682**,686,740,**752**,**806**,810,864,876,**930**,934,988,1000,**1054**,1058,1112,**1124**,**1178**,1182,1236,1248,**1302**,1306,1360,1372,**1426**,1430,1484,1496,**1550**,1554,**1608** | 8,**61**,66,120,132,**185**,190,**243**,256,**309**,314,368,**379**,**433**,438,492,504,**557**,562,**615**,628,**681**,686,740,**751**,**805**,810,864,876,**929**,934,988,1000,**1053**,1058,1112,**1123**,**1177**,1182,1236,1248,**1301**,1306,1360,1372,**1425**,1430,1484,1496,**1549**,1554,**1607** |
|  | Optimal interior knots | 0.00655, **0.03605**, **0.04097**, 0.07045, 0.08356, 0.11303, **0.11798**, 0.14746, 0.16057, **0.19003**, **0.19499**, 0.22447, 0.23758, **0.26704**, **0.27200**, 0.30147, 0.31458, 0.34406, **0.34900**, 0.37848, 0.39159, 0.42108, **0.42601**, 0.45549, 0.46860, **0.49809**, 0.50302, 0.53249, 0.54560, **0.57509**, 0.58002, 0.60950, 0.62261, **0.65209**, **0.65703**, 0.68651, 0.69962, 0.72909, 0.73404, 0.76352, **0.77663**, **0.80609**, **0.81104**, 0.84052, 0.85363, **0.88311**, 0.88805, 0.91753, 0.93064, 0.96012, **0.96506**, 0.99454 | 0.00655, **0.03602**, **0.04098**, 0.07045, 0.08356, 0.11303, **0.11799**, 0.14746, 0.16057, **0.19004**, **0.19500**, 0.22447, 0.23758, **0.26705**, **0.27199**, 0.30147, 0.31458, 0.34406, **0.34901**, 0.37848, 0.39159, 0.42108, **0.42602**, 0.45549, 0.46860, **0.49807**, 0.50302, 0.53249, 0.54560, **0.57510**, 0.58002, 0.60950, 0.62261, **0.65208**, **0.65702**, 0.68651, 0.69962, 0.72909, 0.73404, 0.76352, **0.77662**, **0.80610**, **0.81105**, 0.84052, 0.85363**, 0.88310**, 0.88805, 0.91753, 0.93064, 0.96012, **0.96507**, 0.99454 |
|  | Multiple knot | 3,3,3,3,3,3,3,3,3,3,3,3,3,3,3,3,3,3,3,3,3,3,3,3,3,3,3,3,3,3,3,3,3,3,3,3,3,3,3,3,3,3,3,3,3,3,3,3,3,3,3,3 | 3,3,3,3,3,3,3,3,3,3,3,3,3,3,3,3,3,3,3,3,3,3,3,3,3,3,3,3,3,3,3,3,3,3,3,3,3,3,3,3,3,3,3,3,3,3,3,3,3,3,3,3 |
|  | Fitting error | MSE= 2.3410e-06, ME=0.0060 | MSE= 2.4212e-06, ME= 0.0055 |
|  | Total processing time (ms) | 2490 | 2501 |
| 2 | Spline Degree | $p=3$ | $p=3$ |
|  | Control error | $\epsilon=1e-3$ | $\epsilon=1e-3$ |
|  | Minimum kink angle (degree) | $\alpha_{min}=1$ | $\alpha_{min}=1$ |
|  | Number of pieces at start | $\Omega= 1$ | $\Omega= 20$ |
|  | Maximum smoothness C(k) | $k=-1$ | $k=-1$ |
|  | Number of uniform data in scanning step | $L=10$ | $L=10$ |
|  | Number of loop in Gauss-Newton solving | $M=10$ | $M=10$ |
|  | Scanning knot | *ScanAllFlag =* 1 | *ScanAllFlag =* 1 |
|  | Data bisection time (ms) | 197 | 105 |
|  | Coarse knots by bisection step | 78 knots | 78 knots |
|  | Multiple knot | 52 Triple-knots, 26 single-knots | 52 Triple-knots, 26 single-knots |
|  | Fitting error | MSE= 1.5617e-07, ME=0.0013 | MSE= 6.0987e-08, ME= 0.0012 |
|  | Total processing time (ms) | 3640 | 3715 |
| 3 | Spline Degree | $p=2$ | $p=2$ |
|  | Control error | $\epsilon=1e-3$ | $\epsilon=1e-3$ |
|  | Minimum kink angle (degree) | $\alpha_{min}=5$ | $\alpha_{min}=5$ |
|  | Number of pieces at start | $\Omega= 1$ | $\Omega= 50$ |
|  | Maximum smoothness C(k) | $k=-1$ | $k=-1$ |
|  | Number of uniform data in scanning step | $L=10$ | $L=8$ |
|  | Number of loop in Gauss-Newton solving | $M=10$ | $M=10$ |
|  | Scanning knot | ScanAllFlag = 1 | ScanAllFlag = 1 |
|  | Data bisection time (ms) | 269 | 107 |
|  | Coarse knots by bisection step | 130 knots | 181 knots |
|  | Multiple knot | 78 single-knots, 52 double-knots | 129 single-knots, 52 double-knots |
|  | Fitting error | MSE= 7.4063e-7, ME=0.0018 | MSE= 1.1562e-7, ME= 8.8623e-4 |
|  | Total processing time (ms) | 3970 | 5635 |
