# Supplementary figures and images for: A direct method to solve optimal knots of B-spline curves: An application for non-uniform B-spline curves fitting
Source: PLoS One. 2017 Mar 20;12(3):e0173857. doi: 10.1371/journal.pone.0173857 (PMC5358887; doi:10.1371/journal.pone.0173857)

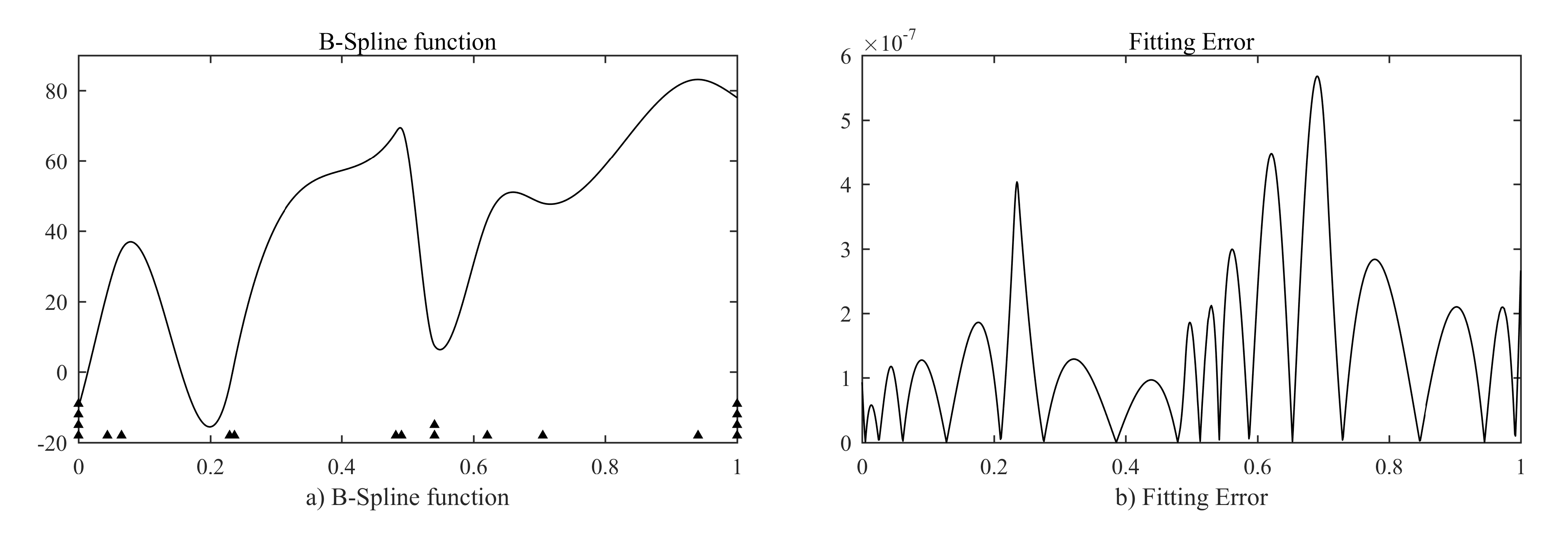

Supplement: S1 File — (ZIP) [file pone.0173857.s006.zip › DemoCode/Fig8.tif]
